# Cryo-EM structures and transport mechanism of human multifunctional transporter BTR1

**DOI:** 10.1093/procel/pwaf108

**Published:** 2025-12-06

**Authors:** Chang Liu, Xudong Chen, Liuliu Chang, Tianyu Li, Zhilin Hao, Ziming Wang, Kun Cui, Chen Zhang, Jingrong Wang, Lulu Guo, Sensen Zhang, Jian Mao, Jianping Xie, Yang Li, Maojun Yang

**Affiliations:** Flavor Science Laboratory, Beijing Life Science Academy (BLSA), Beijing 102209, China; Ministry of Education Key Laboratory of Protein Science, Beijing Advanced Innovation Center for Structural Biology, School of Life Sciences, Tsinghua University, Beijing 100084, China; State Key Laboratory of Drug Research, Shanghai Institute of Materia Medica, Chinese Academy of Sciences, Shanghai 201203, China; University of Chinese Academy of Sciences, Beijing 100049, China; State Key Laboratory of Drug Research, Shanghai Institute of Materia Medica, Chinese Academy of Sciences, Shanghai 201203, China; Flavor Science Laboratory, Beijing Life Science Academy (BLSA), Beijing 102209, China; Flavor Science Laboratory, Beijing Life Science Academy (BLSA), Beijing 102209, China; Flavor Science Laboratory, Beijing Life Science Academy (BLSA), Beijing 102209, China; Flavor Science Laboratory, Beijing Life Science Academy (BLSA), Beijing 102209, China; Flavor Science Laboratory, Beijing Life Science Academy (BLSA), Beijing 102209, China; Flavor Science Laboratory, Beijing Life Science Academy (BLSA), Beijing 102209, China; Flavor Science Laboratory, Beijing Life Science Academy (BLSA), Beijing 102209, China; Flavor Science Laboratory, Beijing Life Science Academy (BLSA), Beijing 102209, China; Flavor Science Laboratory, Beijing Life Science Academy (BLSA), Beijing 102209, China; Ministry of Education Key Laboratory of Protein Science, Beijing Advanced Innovation Center for Structural Biology, School of Life Sciences, Tsinghua University, Beijing 100084, China; State Key Laboratory of Drug Research, Shanghai Institute of Materia Medica, Chinese Academy of Sciences, Shanghai 201203, China; Flavor Science Laboratory, Beijing Life Science Academy (BLSA), Beijing 102209, China; Ministry of Education Key Laboratory of Protein Science, Beijing Advanced Innovation Center for Structural Biology, School of Life Sciences, Tsinghua University, Beijing 100084, China

**Dear Editor,**

The SLC4 transporter family plays a crucial role in maintaining intracellular and extracellular homeostasis. Bicarbonate Transporter Related protein-1 (BTR1), a member of this family, is essential for regulating intracellular pH (Zhang et al., 2015). Initially thought to function as a ${\text{CO}}_{3}^{2-}$ transporter, BTR1 was later identified as an NH_3_/H^+^ co-transporter, a function critical for ammonia metabolism and excretion, supporting kidney, nervous system, and ocular functions (Parker et al., 2001). BTR1 dysfunction is associated with various diseases, including corneal disorders such as congenital hereditary endothelial dystrophy (CHED), Fuchs’ endothelial corneal dystrophy (FECD), and corneal dystrophy and perceptive deafness (CDPD), as well as potential hearing loss, neurological conditions, and metabolic disturbances (Jalimarada et al., 2013; Li et al., 2016; Shimin et al., 2018).

A recent study demonstrated that BTR1 adopts an outward-open conformation when bound to PIP2, while the R125H mutant shifts to an inward-open state, underscoring the role of PIP2 in regulating conformational transitions, pH sensing, and transporter function (Lu et al., 2023). This study also identified non-conserved residues—H719, H724, and P723—as essential for NH_3_/H^+^ co-transport, while the E675Q mutation abolished the H^+^ current, highlighting their functional significance. These findings elucidate the transport mechanism and PIP2-mediated regulation of BTR1, shedding light on disease pathogenesis and potential therapeutic interventions. However, its substrate selectivity and transport dynamics remain unclear, warranting further investigation into its substrate recognition mechanisms and structure–function relationship.

To further elucidate the structural and functional interplay of this protein, we overexpressed human BTR1 in mammalian cells using a Strep affinity tag and reconstituted it in a LMNG/CHS micelle buffer (Fig. S1). Cryo-electron microscopy analysis in a 140 mmol/L KCl buffer at pH 7.5 resolved the BTR1 structure at 3.25 Å (Figs. 1A and S2). The protein forms a homodimer with C2 symmetry, measuring approximately 90 Å in height and 110 Å in width (Fig. 1A–C). The N-terminal sequence constitutes the soluble domain, while the C-terminal sequence forms the transmembrane region (Fig. 1B and 1C). Unlike canonical antiparallel β-sheet structures, β-sheets in each BTR1 monomer interact with those of the opposing monomer, stabilizing the dimer (Fig. 1D). Notably, our cryo-EM structure captured BTR1 in an inward-open conformation, resembling the previously characterized R125H mutant (Fig. 1A).

**Figure 1.**
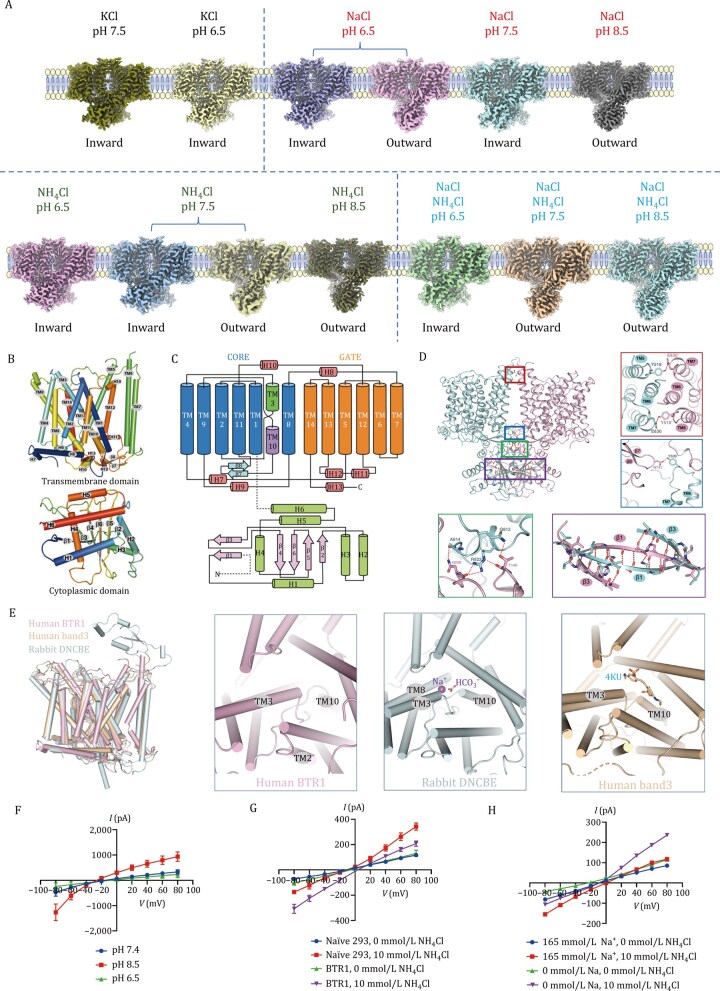
**Structural analysis of BTR1 and electrophysiological characterization**. (A) Cryo-EM structures of BTR1 determined in 13 distinct conformational states under 11 different buffer conditions. The buffer environments are annotated above the schematic membranes, while the corresponding conformational states are indicated below. (B) Cylindrical representation of BTR1, illustrating the transmembrane domain (TMD) and cytoplasmic domain (CD). (C) Ribbon representation of the BTR1 dimer interface. The red and blue boxes highlight interactions between the transmembrane domains (TMDs), while the green and purple boxes indicate interactions between the cytoplasmic domains (CDs) and between the TMD and CD, respectively. (D) Analysis of the dimer interface in the inward-open conformation of BTR1, with different colored boxes representing distinct interaction sites. (E) Structural comparison of the transmembrane domains (TMDs) and substrate/small-molecule binding sites among human BTR1 (in inward-open conformation), rabbit NDCBE (PDB: 7RTM), and human Band 3 (PDB: 4YZF). (F) Patch-clamp recordings of macroscopic currents in BTR1-overexpressing cells with 10 mmol/L NH_4_Cl at different pH levels. (G) patch-clamp recordings of macroscopic currents in naïve HEK293T cells and BTR1-overexpressing cells, in the presence or absence of 10 mmol/L NH_4_Cl. (H) Patch-clamp recordings in BTR1-overexpressing HEK293T cells under different ionic conditions: 165 mmol/L NaCl (with and without 10 mmol/L NH_4_Cl), Na^+^-free conditions, and NH_4_Cl-only conditions.

Unlike other SLC4 family proteins (Fig. S3), the soluble domain of BTR1 is tightly integrated with its transmembrane region, suggesting a unique regulatory function. The intracellular domain of BTR1 is structurally distinct from those of NDCBE (Wang et al., 2021) and Band3 (Arakawa et al., 2015) (Figs. S4 and S5). While BTR1 and NDCBE both form symmetric dimers, their dimerization patterns and conformations diverge (Wang et al., 2021). In BTR1, intracellular domains exhibit greater β-sheet interactions compared to NDCBE (Fig. S5B and S5C). In contrast, Band3 dimerizes asymmetrically, further distinguishing its structure from BTR1 (Fig. S5B and S5D). These structural differences suggest that BTR1 operates via a unique ion-binding mode (Fig. 1E) and regulatory mechanism compared to other SLC4 transporters (Thurtle-Schmidt and Stroud, 2016). Further structural analysis of the apo BTR1 dimer identified key interactions at the dimer interface, including contacts between monomers at the membrane (red and blue boxes), within the intracellular domain (purple box), and between the transmembrane and intracellular regions (green box) (Fig. 1D).

Previous studies suggest a voltage-dependent activation feature of BTR1 (Meeyoung et al., 2014). To determine BTR1’s potential role in boric acid transport, we conducted electrophysiological experiments in both wild-type HEK293T cells and BTR1-expressing cells. No boric acid-induced currents were detected in either, confirming its lack of boric acid transport activity (Figs S6 and S7). We then examined proton transport capacity and pH-dependent structural changes of BTR1. Cryo-EM analysis of BTR1 at pH 6.0, in the absence of sodium ions, yielded structures at resolution of 2.77 Å (Fig. S8). Similar to the structure determined at pH 7.5, BTR1 at pH 6.0 also adopts an inward-facing apo conformation, indicating that pH fluctuations alone are insufficient to induce significant conformational changes or activation (Fig. 1A).

Next, we examined whether sodium ions function as substrates for BTR1 by analyzing its conformation in sodium chloride solutions at pH 6.5, 7.5, and 8.5 ­(Figs. S9–11). At neutral pH, BTR1 maintained an inward-facing conformation. Under acidic conditions (pH 6.5), two conformational states were observed: inward-­facing and outward-facing (Fig. 1A), suggesting sodium ions as substrates for BTR1. In contrast, at alkaline pH (pH 8.5), BTR1 fully transitioned to an outward-facing conformation, consistent with prior studies (Lu et al., 2023). Additionally, PIP2 was detected within the transmembrane regions (Fig. S12A). Electrophysiological experiments showed that BTR1 has transport activity under both acidic and alkaline conditions (Fig. 1F). This interaction enables pH-dependent conformational transitions, where alkaline pH favors the outward-facing state.

To determine whether BTR1 transports NH_3_ or ${\text{NH}}_{4}^{+}$, we analyzed its conformation in ammonium chloride solutions at pH 6.5, 7.5, and 8.5 in the absence of sodium ions. Due to ${\text{NH}}_{4}^{+}$ ionization, the NH_3_ concentration in solution varies with pH: 0.017 mmol/L at pH 6.5, 0.17 mmol/L at pH 7.5, and 1.47 mmol/L at pH 8.5. If ${\text{NH}}_{4}^{+}$ were the transport substrate, BTR1 would exhibit activation across all pH conditions. In contrast, if NH_3_ were the substrate, only solutions containing at least 0.17 mmol/L NH3 should activate BTR1, which was observed in our electrophysiological experiments (Fig. 1G). Cryo-EM analysis revealed that at pH 6.5, BTR1 remained in an inward-facing conformation (Fig. S13), while it exhibited both inward- and outward-facing states at pH 7.5 (Fig. S14). At pH 8.5, BTR1 fully transitioned to an outward-facing conformation (Fig. S15). These findings indicate the influence of ammonium chloride toward the conformation of BTR1, with higher NH_3_ concentrations promoting the outward-facing state. Electrophysiological experiments further confirmed the role of BTR1 in NH_3_ transport (Fig. 1G). Applying 10 mmol/L NH_4_Cl to non-transfected HEK293T cells induced an outward current, whereas BTR1-overexpressing cells exhibited a decreased outward current and an increased inward current. Current–voltage relationship analysis showed that BTR1 expression had minimal impact under NH_4_Cl-free conditions but caused significant changes when NH_4_Cl was present, confirming NH_3_ as a potent activator of BTR1.

Next, we investigated whether sodium ions facilitate NH_3_ transport (Fig. S16). Structural analysis of BTR1 in sodium chloride and ammonium chloride solutions at both neutral and alkaline pH conditions induced an outward-facing conformation (Fig. 1A). In neutral pH, the addition of sodium chloride shifted BTR1 from a mixed to an outward-facing conformation, indicating that sodium ions enhance ammonia transport. Electrophysiological experiments further demonstrated a synergistic effect between sodium and NH_3_ transport (Fig. 1H). Under sodium-free extracellular conditions, BTR1-mediated NH_4_Cl currents lost their inward rectification, resulting in a linear current–voltage relationship.

The electrophysiological and structural studies presented above suggest that BTR1 can transport sodium ions, H^+^, OH^–^, and NH_3_. To further characterize BTR1’s substrate specificity and conformational transitions, we analyzed BTR1 across 11 buffer environments with varying pH levels in KCl, NaCl, NH_4_Cl, NH_4_Cl + NaCl, and boric acid, identifying 13 distinct BTR1 states, predominantly in inward-facing and outward-facing conformations (Fig. 1A). Consistent with previous findings, we observed substrate-like densities in different conformations ­(Fig. S12B and S12C), though precise identification was not available. To gain deeper insights into transport capacity of BTR1, we employed membrane-stretching molecular dynamics simulations, initiating the process from the outward-facing conformation to examine Na^+^, H^+^, and NH_3_ transport. We assessed changes in potential energy, hydrogen bonding, root mean square deviation (RMSD), and solvent-accessible surface area (SASA) over time, and evaluated their influence on protein structure. Stability analyses indicated that all substrate-bound complexes remained stable during stretching. Among them, Na^+^ exhibited the highest potential energy (−80.61 ± 0.05 × 10^4^ kcal/mol), followed by H^+^ (−81.88 ± 0.05 × 10^4^ kcal/mol) and NH_3_ (−82.19 ± 0.05 × 10^4^ kcal/mol) (Fig. 2A). Hydrogen bond analysis revealed that NH_3_ induced the most significant alterations, likely due to its ability to form hydrogen bonds modulating the hydrogen bond network within the protein during transport (Fig. 2B). SASA analysis showed that H^+^ had minimal impact, consistent with its small size, whereas NH_3_ and Na^+^ increased the surface exposure of the protein, correlating with RMSD data indicating more pronounced conformational changes for these substrates (Fig. 2A and 2B). During transport, NH_3_ and Na^+^ triggered notable structural rearrangements, leading to system expansion and increased SASA.

**Figure 2.**
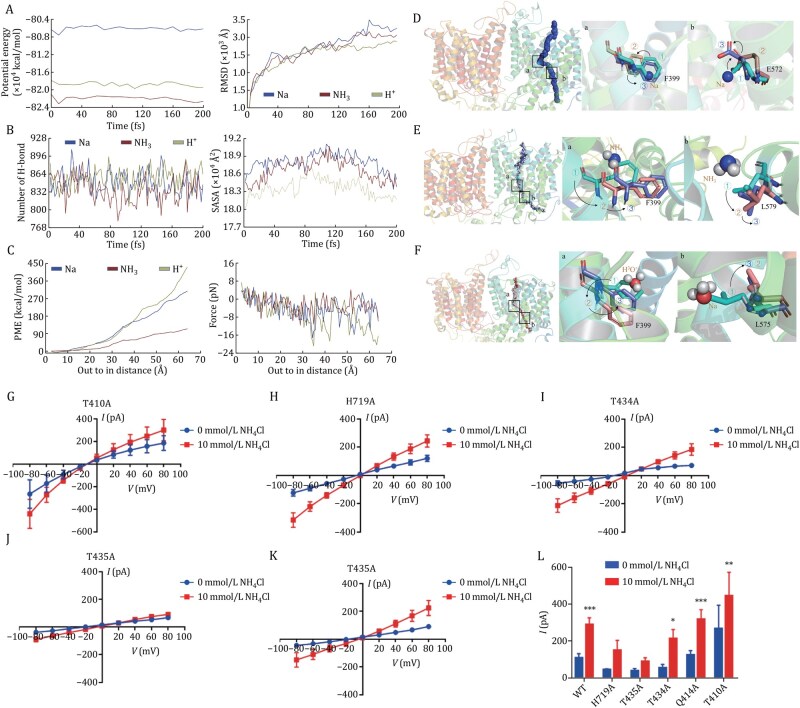
**Binding interactions and transport dynamics of Na^+^, NH_3_, and H^+^ in BTR1**. (A) Time-dependent potential energy profiles, with mean values of −80.61 ± 0.05, −82.19 ± 0.05, and −81.88 ± 0.05 × 10^4^ kcal/mol, respectively. Root-mean-square deviation (RMSD) analysis over time for three simulated systems. (B) Changes in the number of hydrogen bonds during substrate translocation. Solvent-accessible surface area (SASA) of the three complex volumes as a function of time. (C) Potential of mean force (PMF) as a function of stretching distance. Force variation along the stretching distance. (D) Motion pattern analysis of Na^+^ during ASMD simulations, showing in inward conformation. (E) Motion pattern analysis of NH_3_ during ASMD simulations, showing in inward conformation. (F) Motion pattern analysis of H_3_O^+^ during ASMD simulations, showing in inward conformation. (G–L) Voltage–current relationships in HEK293T cells overexpressing BTR1 mutants (T410A, Q414A, T434A, T435A, H719A) in the presence or absence of 10 mmol/L NH_4_Cl. Two-way ANOVA followed by the Sidak test was used for statistical analysis. The data are shown as the mean ± SEM, * indicates *P *< 0.05, ** indicates *P *< 0.01, *** indicates *P *< 0.001, compared with 0 mmol/L NH_4_Cl.

To further probe substrate translocation, we analyzed the Potential of Mean Force (PMF) during molecular dynamics simulations. The results demonstrated a substantial force is required for substrates to enter the active site from the extracellular environment, overcoming steric hindrance imposed by small pore sizes and multiple transport channels (Fig. 2C). PMF analysis revealed that NH_3_ required the lowest average force, while Na^+^ and H^+^ exhibited higher values, potentially due to the ability of NH_3_ to form stabilizing hydrogen bonds with the protein (Fig. 2C). Once inside the transport pocket, substrate movement to the membrane interior followed distinct patterns. H^+^ traversed freely without additional energy input, whereas NH_3_ and Na^+^ encountered minor resistance, likely due to steric hindrance from surrounding residues. Traction force analysis confirmed H^+^ as the most readily transported substrate, while NH_3_ exhibited that the most significant resistance. Structural analysis revealed Na^+^ binding triggered a conformational change in F399, which shifted backward to facilitate Na^+^ passage and subsequently returned to its original position to prevent backflow. Similarly, upon Na^+^ exit from the active pocket, the side chain of E572 reoriented to open the transport channel, allowing Na^+^ to enter the inner membrane region (Fig. 2D). For NH_3_ transport, F399 also underwent a conformational shift, while L579 moved outward to accommodate NH_3_ passage (Fig. 2E). In proton transport, F399 returned to its initial position to maintain selectivity, whereas L575 moved inward to stabilize the inner state and facilitate H_3_O^+^ entry into the inner membrane channel (Fig. 2F). Collectively, PMF, traction force, and transport pathway analyses suggest a hierarchy of transport efficiency: H^+^ > Na^+^ > NH_3_.

To identify key residues involved in substrate binding, we analyzed various BTR1 conformations and pinpointed critical amino acid interactions within the substrate-binding cavity (Fig. 2G–I). To validate their functional significance, we introduced targeted mutations and assessed their effects using electrophysiological experiments (Figs. 2L and S17). Compared to wild-type BTR1, Q414A and T410A mutants exhibited significant transport differences in the presence or absence of NH_4_Cl (Fig. 2G and 2H). In contrast, H719A, T434A, and T435A mutations reduced current responses (Fig. 2I–K). Among them, T434A and T435A displayed transport currents nearly identical to blank cells, indicating their essential role in NH_3_ transport. The functional similarity between T434A and T435A likely arises from their spatial proximity and shared physicochemical properties. Additionally, the H719A mutation produced current characteristics similar to NH_3_ transport by BTR1 in sodium-free conditions, suggesting a crucial role for H719 in Na^+^ binding.

Our findings establish BTR1 as a unique member of the SLC4 family with dual functional modes (Guha and Roy 2021). In the absence of NH_3_/${\text{NH}}_{4}^{+}$, BTR1 operates as a Na^+^-dependent [H^+^]/[OH^–^] transporter, activated under acidic conditions. However, in the presence of NH_3_/${\text{NH}}_{4}^{+}$, particularly NH_3_, BTR1 functions as an NH_3_ transporter, with Na^+^ enhancing its transport efficiency. Structural and sequence analyses reveal that BTR1 undergoes a distinctive transport-associated conformational shift, characterized by minimal rearrangement in the transmembrane domain but significant rotational and elevation changes in the cytosolic region—a phenomenon not observed in other SLC4 family proteins.

In conclusion, by integrating structural and functional analyses, we delineate the transport mechanism of BTR1 and propose a novel mode of action. Our study expands the understanding of substrate recognition and transport mechanisms within the SLC4 family, providing new insights into human acid-base balance (Boudker and ­Verdon, 2010; Damkier et al., 2007).

## Supplementary Material

pwaf108_Supplementary_Data

## Footnotes

This work was supported by the National Key R&D Program of China (2022YFA1302701), the National Natural Science Foundation of China (Nos. 32272455, 32030056, 32571390), the Scientific Project of Beijing Life Science Academy (Nos. 2023000CB0010, 2023300CA0090, 2024300CC0180, 2025300CB0030, and 2024301QPIA01), and China National Tobacco Corporation (No. 110202404020).

All authors declare no conflict of interests. All authors have read and approved the manuscript for publication.

All relevant data and materials in this article are available upon reasonable request from the lead contact.

M.Y. and Y.L. directed the study. C.L., X.C., and S.Z. did the protein purification. X.C. and S.Z. performed EM sample preparation, data collection and structural determination with the help of L.H., Z.W., and K.C.; L.C. and T.L. performed the electrophysiological experiments with the help of C.Z., J.W., and L.G.; M.Y., X.C., and S.Z. built the model and drew the figures. M.Y., J.X., J.M., and S.Z. wrote the manuscript. All authors contributed to the discussion of the data and edited the manuscript. Cryo-EM density maps and coordinates generated in this study have been deposited in the Electron Microscopy Data Bank (EMDB) and the Protein Data Bank (PDB) with the following accession numbers: BTR1-NaCl_pH6.5_Inward (EMD-67782 and PDB-21KY); BTR1-NaCl_pH6.5_Outward (EMD-67783 and PDB-21KZ);BTR1-KCl_pH7.5_Inward (EMD-67784);BTR1-KCl_pH6.5_Inward (EMD-67785);BTR1-NaCl_pH7.5_Inward (EMD-67786);BTR1-NaCl_pH8.5_Outward (EMD-67787);BTR1-KCl_NH_4_Cl_pH6.5_Inward (EMD-67788);BTR1-KCl_NH_4_Cl_pH7.5_Inward (EMD-67791);BTR1-KCl_NH_4_Cl_pH7.5_Outward (EMD-67792);BTR1-KCl_NH_4_Cl_pH8.5_Outward (EMD-67793);BTR1-NaCl_NH_4_Cl_pH7.5_Outward (EMD-67794);BTR1-NaCl_NH_4_Cl_pH8.5_Outward (EMD-67795);BTR1-NaCl_NH_4_Cl_pH6.5_Inward (EMD-67796).
