# Supplementary material for: Cryo-EM structures and transport mechanism of human multifunctional transporter BTR1
Source: Protein Cell. 2025 Dec 6;17(4):366–71. doi: 10.1093/procel/pwaf108 (PMC13107553; doi:10.1093/procel/pwaf108)
Supplement: pwaf108_Supplementary_Data [file pwaf108_supplementary_data.pdf]

Methods

Materials

The following reagents were purchased from Sigma-Aldrich: NaCl (S9888), KCl (239313), NH_4Cl (A9434) , boric acid (B2768), HEPES(4-(2-Hydroxyethyl)piperazine-1-ethanesulfonic acid; H3375).In addition, protease inhibitor cocktail tablets (04693116001) were obtained from Roche. The detergent 10:1 LMNG/CHS pre-made solution (NG310-CH210) and digitonin (D3203) were obtained from Anatrace. Strep-Tactin resin (2-1208-500) and D-desthiobiotin (2-1000-005) were ordered from IBA. For cell culture, human embryonic kidney (HEK) 293F cells (R79007) were purchased from Thermo Fisher Scientific , the cell culture medium (SMM 293-TII) were obtained from Sino Biological and the penicillin-streptomycin solution (SV30010) were ordered from Hyclone. We obtained the linear polyethylenimine (PEI, MW 25000; 23966) from Polysciences.

Cell culture and transfection

The optimized coding cDNA for *Homo sapiens BTR1* (Uniprot: Q8NBS3-1) including a C-terminus tandem twin Strep-tag was cloned into the pCAGGS vector for mammalian expression. HEK293F cells were cultured in medium supplemented with 1× penicillin/streptomycin in a Multitron-Pro shaker (Infors, 120 rpm) at 37°C with 5% CO_2.To produce intact BTR1 proteins, 1mg plasmids was pre-incubated with 2.5 mg PEI in 50 mL fresh medium for 25 minutes prior to adding the mixture to one-liter cells when cell density reached 1.6×10^6 per milliliter. The transfected cells were cultured for 48 h before harvesting.

Protein expression and purification for cryo-EM analysis

In order to obtain BTR1 protein in different conformational states, in total 12 batches of protein purifications were performed. For one batch of protein purification, about six liters of transfected cells were harvested by centrifugation at 3,000×*g*. All procedures below are carried out at 4 °C or on ice. To purify BTR1 without Na^+ in natural condition, harvested cells were resuspended in lysis buffer containing 25mM HEPES (pH 7.5), 150 mM KCl, 2mM DTT, and protease inhibitor, and then lysed by high-pressure homogenizer. After removal of cell debris by centrifugation at 10,000 ×*g* for 45 minutes, cell membrane fraction was pelleted by a 150,000 ×*g* ultracentrifugation for 1 hour. The membrane fraction was resuspended and solubilized in lysis buffer plus 1% (w/v) digitonin for 2 hours with gentle rotation. After

ultracentrifugation at 150,000 ×*g* for 30 minutes, the supernatant was passed through a column filled with Strep-Tactin Sepharose resin. The resin was washed 50 CV (column volume) with wash buffer containing 25mM HEPES (pH 7.4), 150 mM KCl, 2mM DTT, 0.1% digitonin . The target BTR1 protein was eluted with wash buffer plus 10 mM desthiobiotin. The eluted BTR1 protein was concentrated to a final volume of approximately 100 µl by a 100 kDa cut-off centrifugal filter (Millipore) and further purified by SEC (size-exclusion chromatography) (Superose 6 5/150, GE Healthcare) in SEC buffer containing 25mM HEPES (pH 7.5), 150 mM KCl, 0.1% (w/v) digitonin. The SEC fractions corresponding to BTR1 were collected and verified by SDS-PAGE for Cyro-EM sample preparation (Extended Data Fig. 1). The peak fractions were concentrated to 15 mg ml^{-1} for grid preparation. Similar protocol with corresponding modifications were used to purify BTR1 in different state or complex with different substrates . In brief, the concentration of HEPES and detergent was the same, however, the type of the salt and the pH of the buffer were made corresponding changes during each batch of protein purification.

Electron microscopy sample preparation and imaging

The cryo-EM grids were prepared using Vitrobot Mark IV (FEI) at 8°C and 100% humidity. 4 µL aliquots of samples at a concentration of 15 mg ml^{-1} were applied onto glow-discharged holey carbon grids (Quantifoil R1.2/1.3). After a waiting time of 5 s, the grids were blotted for 2–5 s and plunged into liquid ethane for quick freezing. The grids were screened on a Tecnai Arctica microscope (FEI) operated at 200 kV using a Falcon II direct electron detector (FEI). For the cryo-sample of BTR1 without Na^+ in acidic condition, the qualified grids were transferred into a Titan Krios microscope (FEI) operated at 300 kV equipped with a Cs image corrector and an energy filter (slit width 20 eV; GIF Quantum LS, Gatan) for data acquisition. Images were recorded using a K3 submit direct electron detector (Gatan) in a super mode at a nominal magnification of 64, 000 ×, corresponding to a calibrated pixel size of 0.54895 Å. Date acquisition was performed automatically using AutoEMation2 (Lei and Frank, 2005) in a movie mode, with a frame exposure time of 0.08 s and a total exposure time of 2.56 s, resulting in a total of 32 frames per stack and the total dose for each stack was approximately 50 e^{-} $Å^{-2}$. All 32 frames in each stack were aligned and summed using the whole-image motion correction program MotionCor2 [1] and binned to a pixel size of 1.0979.

For the other BTR1 cryo-samples, the qualified grids were transferred into a Titan Krios microscope (FEI) operated at 300 kV equipped with an energy filter (slit width 20 eV; GIF Quantum LS, Gatan) for data acquisition. Images were recorded using a K3 submit direct electron detector (Gatan) in a super mode at a nominal magnification of 105, 000 ×, corresponding to a calibrated pixel size of 0.4187 Å. Date acquisition was performed automatically using AutoEMation2 in a movie mode, with a frame exposure time of 0.08 s and a total exposure time of 2.56 s, resulting in a total of 32 frames per stack and the total dose for each stack was approximately 50 e^{-} $Å^{-2}$. All 32 frames in each stack were aligned and summed using the whole-image motion correction program MotionCor2 and binned to a pixel size of 0.8474.

Image processing and 3D reconstruction

For the cryo-EM dataset of the BTR1 protein under different conditions, a specific number of micrographs were collected for each state. Image processing was performed using cryoSPARC[2]. For the data set of BTR1 without Na^{+} in natural condition, micrographs were imported and the CTF corrections were performed using Patch CTF estimation(Extended Data Fig. 2). After the deletion of bad micrographs, 200 micrographs were used for automatic picking by blob picker, and these particles were subjected to 2D classification. The class averages representing projections of the BTR1 in different orientations were chosen as templates for template picking from the whole dataset. A total of 2,528,276 particles were picked from 2,302 micrographs. These particles were extracted and binned 3 times and subjected to 2D classification. After 3 rounds of 2D classification, ~552K particles in good 2D averages were picked and subjected to the ab-initio reconstruction and the following heterogenious refinement. The map of each class were measured in Chimera(Eric et al., 2008), and the particles from good classes were chosen and re-extrated to the original pexel size of 0.8374 Å. These particles were then subjected to next round of ab-initio reconstruction and heterogenious refinement to further remove bad particles. After 2 rounds of this operation, the final data set of 112,763 particles from 4 classes were used for 3D reconstruction by non-uniform (NU) refinement(Punjani et al., 2020) containing the local and global CTF refinement to yield a map at 3.25-Å resolution map with C2 symmetry imposed. The local resolution map was calculated using local resolution estimation and displayed in ChimeraX(Goddard et al., 2017). Similar data processing

procedures were applied for the other data sets of BTR1.

Model building

The ten BTR1 density maps at 2.50-3.50 resolution were all of sufficient quality for *de novo* model building in COOT(Emsley and Cowtan, 2004), facilitated by the structure of human full-length BTR1 prediced on AlphaFold(Jumper et al., 2021;Punjani et al., 2020). For model building, the predicted model of human BTR1 was docked into the BTR1 without Na^+ in acidic condition cryo-EM map with a resolution of 2.77 Å in Chimera and manually adjusted in COOT to acquire the atomic model of BTR1 dimer(Emsley et al.2010). Model refinement was performed on the main chain of the two atomic models using the real_space_refine module of PHENIX[3](Adams et al.2002) with secondary structure and geometry restraints to avoid over-fitting. After manual adjustment in COOT, the models were subjected to real-space refinement in PHENIX. For model buildings of the other nine BTR1 maps, similar procedure was used with the help of the new acquired BTR1 model.

Site-Directed mutagenesis

Site-directed mutagenesis of the pCAGGS-*BTR1* plasmid was constructed using Mut Express II Fast Mutagenesis Kit (Vazyme Biotech). The resulting pCAGGS-*BTR1*-(T410A, Q414A, T434A, T435A or H719A) plasmid DNAs were isolated using the QIAprep Spin Miniprep Kit (Qiagen) and verified by sequence analysis.

Electrophysiology

All electrophysiological recordings were obtained using patch-clamp recordings for macroscopic current of BTR1 and its mutants over-expressed in HEK293T cells. Patch-clamp recordings were conducted 24−48 h after transfection. The bath solution contained 150 mM NaCl, 10 mM HEPES, pH 7.4. The pipette solution contained 140 mM KCl, 10 mM HEPES, pH 7.2. Patch pipettes were pulled from PC-10 (NARISHIGE) and heat polished to a resistance of 3–10MΩ. Patch-clamp recordings were performed with Axopatch-200B amplifier and Axon Digidata 1550A driven by Clampex10 software (Molecular Devices). The current signals were filtered at 1 kHz and digitized at a 10 kHz sampling frequency. The holding potential was set to 0 mV. The whole cell currents were recorded by voltage steps from -80 mV to +80 mV in 20 mV increments. Liquid junction potentials were less than 3 mV calculated by JPCalc software. All values were measured using Clampfit 10.6 Software.

Statistical analysis

Statistical analyses, including Two-way ANOVA, were conducted using GraphPad Prism software. All statistical data are reported as mean ± standard error of the mean (s.e.m.). $P < 0.05$ was considered statistically significant.

Molecular dynamics of membrane stretching

In order to verify the difference in the ability of the three small molecules(H_3O^+,NH_3,Na^+) to pass through the SLC4A11/BTR1 protein, a membrane MD(Molecular Dynamics) simulation of 10ns was first performed for the complex structure, and then a tensile MD was completed based on the membrane MD simulation.The MD simulation was performed using the AMBER20 software package[3](Case et al.2018) and the ff16SB force field[3](Simmerling et al., 2015), and the solvent model was used as the TIP3P water model[3,4](Jorgensen et al., 1983). The complex systems (substrates Na, NH_3, and protons) were supplemented with 80977/82706/82206 water molecules, 80/80/80 POPCs, 80/80/80 POPEs, and 40/40/40 POPGs, respectively. Two-step energy optimization of the three systems was performed prior to the MD simulations: (1) Solute constraints (constraint constants of 2.09×10^5 $kJ{\cdot}mol^{-1}{\cdot}nm^{-2}$), 5000 steps of steepest descent and 5000 steps of conjugate gradient optimization were performed. (2) Unconstrained steepest descent and 5000-step conjugate gradient optimization, with the convergence condition set to an energy gradient of less than 4.182×10^{-4} $kJ{\cdot}mol^{-1}{\cdot}nm^{-2}$. After the energy optimization, the MD simulation also consisted of two steps:(1) MD simulation with 5ns solute constraints, with a constraining force constant of 41.82 $kJ{\cdot}mol^{-1}{\cdot}nm^{-2}$, during which the temperature of the system was gradually increased from 0 K to 300 K. (2) Unconstrained constant-temperature MD simulation with 5ns, with the SHAKE algorithm[5] to constrain the bond length of hydrogen-containing atoms, and the radius of nonbonding interactions was set to 8 Å. The integration step was set to 2 fs, and the conformations were collected at 1 ps intervals, with a total of 1,000 conformations collected for the subsequent analysis of the results.

In the state of obtaining a stable structure, the processes of the three substrates in and out of the transporter channel are discussed by stretching molecular dynamics. In order to minimize the effect of external forces on the substrate and protein conformation, the pulling process in

the ASMD simulation was first performed with the Cα atom in SLC4A11/BTR1 Pro620 and the Na/N/O atoms in the substrate immobilized and pulled, respectively. A constant pulling speed of 100 Å/ns and a harmonic force constant k of 2.5 kcal/mol·$Å^2$ were used to increase the distance from the outer end of the membrane to the inner end of the membrane by 65 Å, resulting in a slow pulling process. The pulling process was averaged into 5 phases, each producing 25 trajectories, to obtain statistically reliable results. All ASMD simulations were also performed through the PMEMDGPU implementation in the AMBER20 software package, data acquisition was performed at a time step of 2 fs under the NVT set and the PMF was calculated along the traction coordinates using a Langevin thermostat kept at 300 K.

Homology modeling

The modeling was done in Discovery Studio (DS) 2.5. Based on the target sequences, sequence comparison was performed by Align Multiple Sequence module; homology modeling was performed by MODELER module; and finally, the protein structure was evaluated by Ramachandran plot parameter, which can visualize the permissible conformations of amino acid residues in the modeled structure. The more residues fall in the allowed conformational region, the higher the confidence of the structure, and all parameters are default values in the calculation process.

Extended data figures and Figure legends

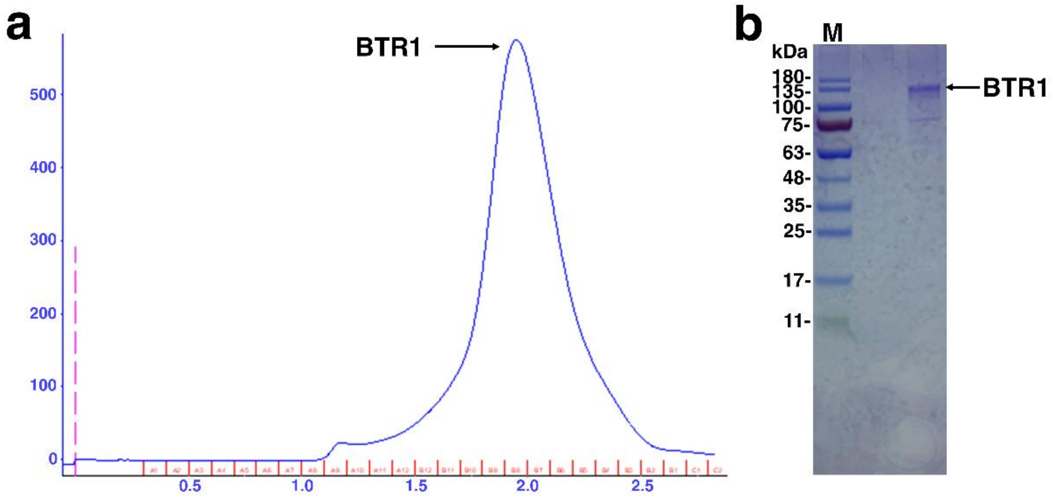

Extended Data Fig. 1 Protein purification of human BTR1

a, A representative trace of size-exclusion chromatography of human full-length BTR1 without Na^+ in natural condition by Superose 6 5/150 GL column. UV, ultraviolet.

b, The peak frections of protein samples in **a** were subjected to SDS-PAGE.

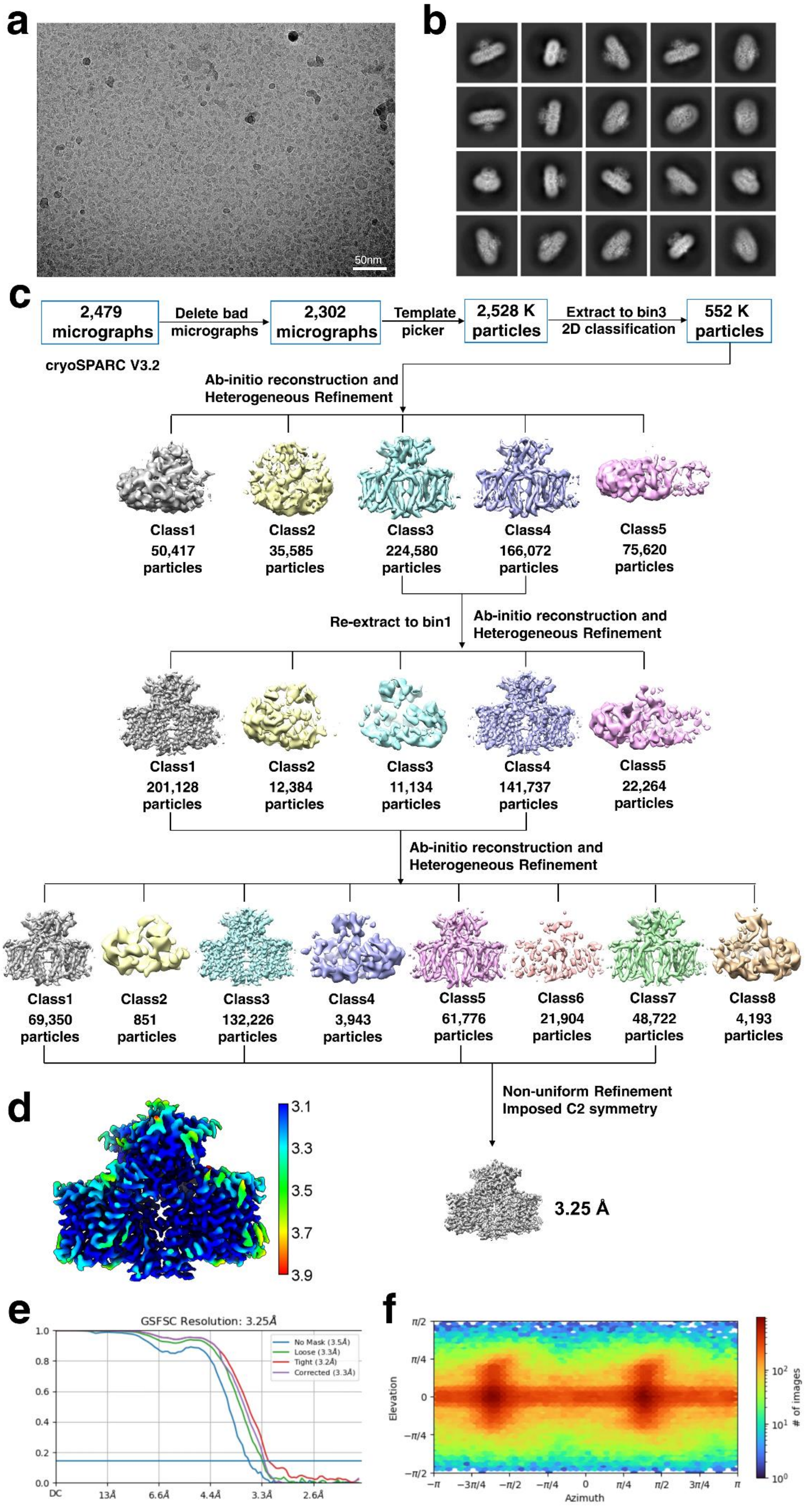

a
50nm
b
c
2,479 micrographs
Delete bad micrographs
2,302 micrographs
Template picker
2,528 K particles
Extract to bin3 2D classification
552 K particles
cryoSPARC V3.2
Ab-initio reconstruction and Heterogeneous Refinement
Class1 50,417 particles
Class2 35,585 particles
Class3 224,580 particles
Class4 166,072 particles
Class5 75,620 particles
Re-extract to bin1
Ab-initio reconstruction and Heterogeneous Refinement
Class1 201,128 particles
Class2 12,384 particles
Class3 11,134 particles
Class4 141,737 particles
Class5 22,264 particles
Ab-initio reconstruction and Heterogeneous Refinement
Class1 69,350 particles
Class2 851 particles
Class3 132,226 particles
Class4 3,943 particles
Class5 61,776 particles
Class6 21,904 particles
Class7 48,722 particles
Class8 4,193 particles
Non-uniform Refinement Imposed C2 symmetry
d
3.1
3.3
3.5
3.7
3.9
3.25 Å
e
GSFSC Resolution: 3.25Å
No Mask (3.5Å)
Loose (3.3Å)
Tight (3.2Å)
Corrected (3.3Å)
DC 13Å 6.6Å 4.4Å 3.3Å 2.6Å
f
Elevation
Azimuth
# of images

Extended Data Fig. 2 Reconstruction and structure determination of human BTR1 without Na^+ in natural condition.

a,b Representative cryo-EM micrograph (**a**) and 2D class averages (**b**) of human BTR1 without Na^+ in natural condition.

c, The workflow of cryo-EM data processing by cryoSPARC[6].

d, Local resolution map of the final 3D density map.

e, Gold-standard Fourier Shell correlation (FSC) curve after 3D refinement. The resolution estimation was based on the criterion of FSC 0.143 cutoff.

f, Particle orientation distributions in the last iteration of the structural refinement.

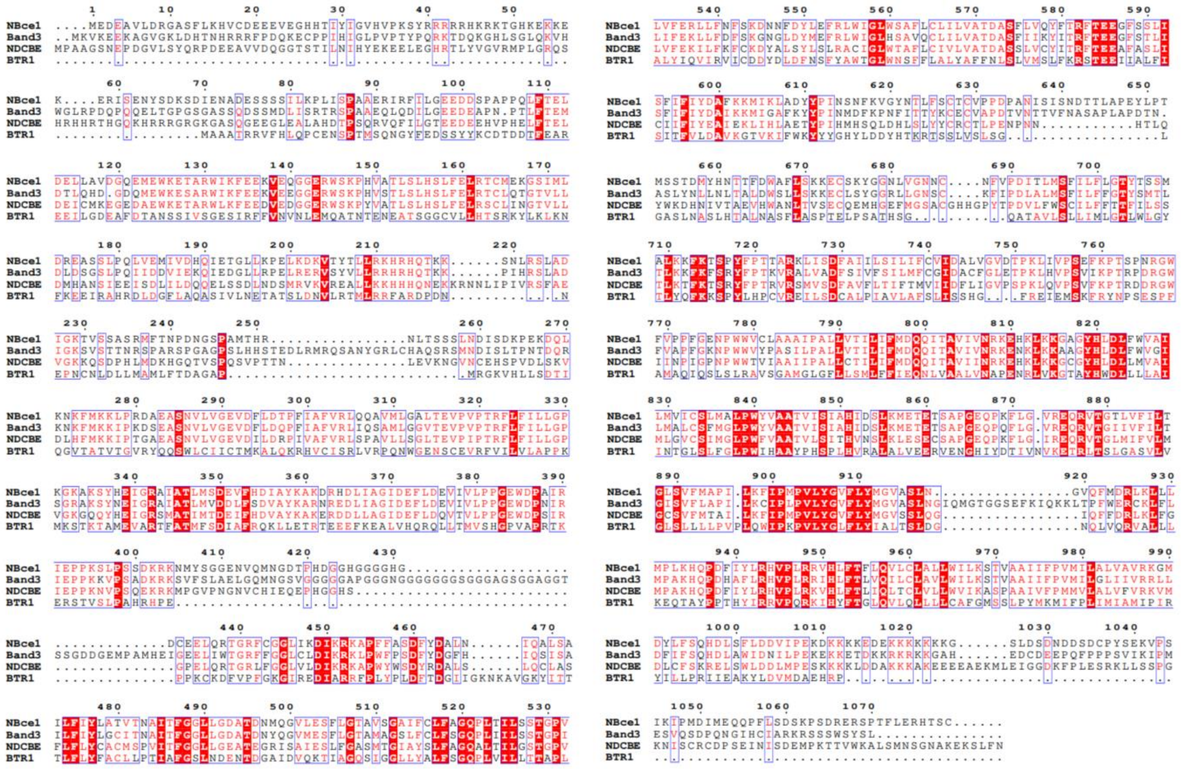

Extended Data Fig. 3 Sequence alignment of SLC4 family proteins

a, Sequence alignment of BTR1, NBCe1, NDCBE, and Band3. Residues are considered as highly similar are colored in red and framed in blue.

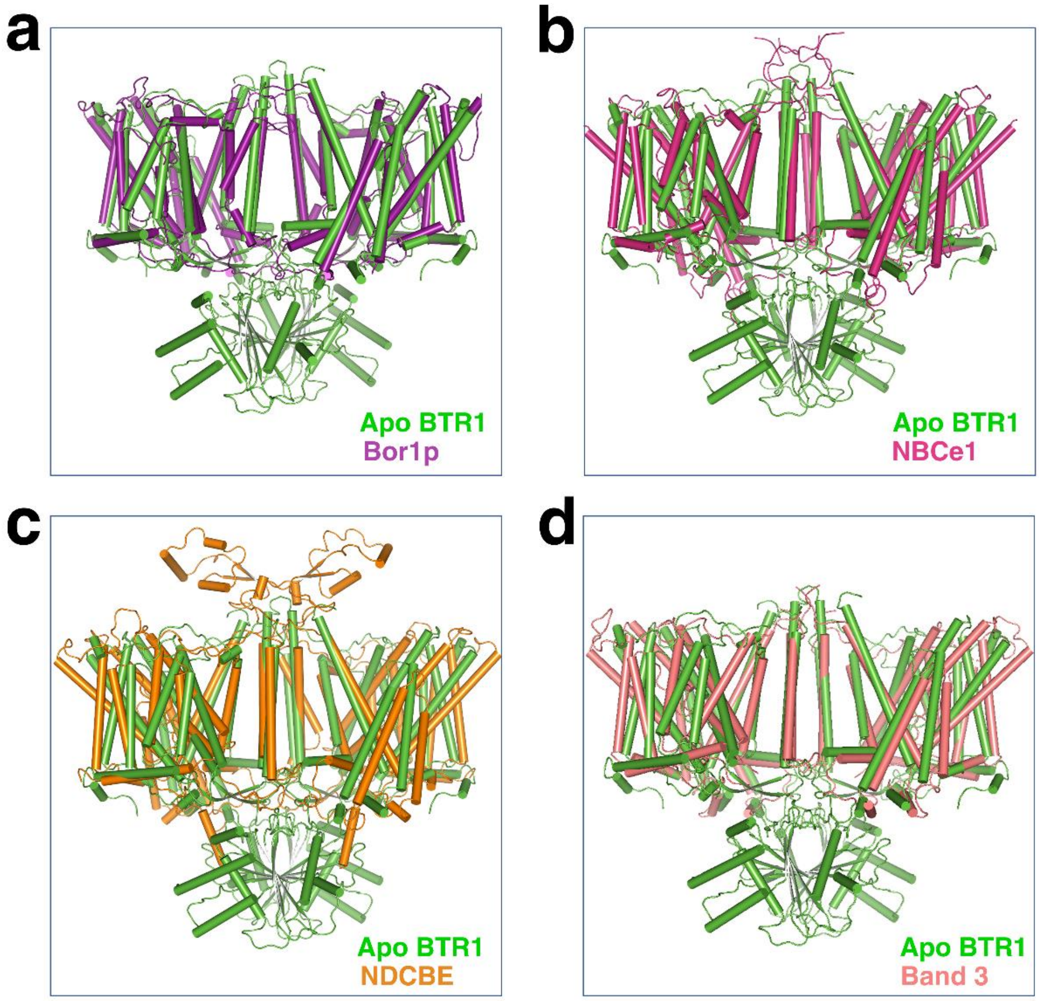

Extended Data Fig. 4 Structural comparisons of human BTR1 in apo state with other SLC4 family protein.

a-d, Structural comparison of human BTR1 in apo state (green) and four SLC4 models PDB: 5SV9 (**a**, purple), 6CAA (**b**, hotpink), 7RTM (**c**, orange) and 4YZF (**b**, salmon).

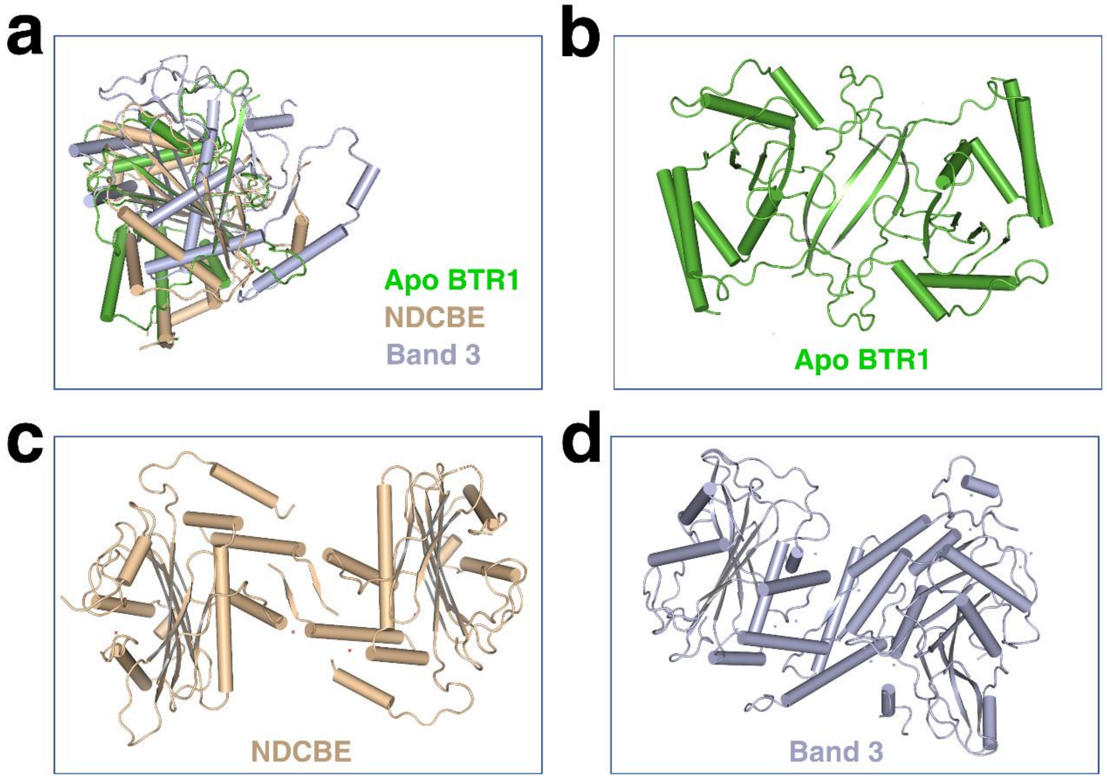

Extended Data Fig. 5 Structural comparisons of the cytosolic domain of human BTR1 in apo state with other SLC4 family protein.

a, Structural comparison of the monomeric cytosolic domain of human BTR1 in apo state (green) and two SLC4 models PDB: 5JHO (wheat) and 8CSY (light blue).

b-d, Structures of the dimeric cytosolic domain of BTR1 (green), NDCBE (PDB: 5JHO, wheat) and Band 3 (PDB: 8CSY , light blue).

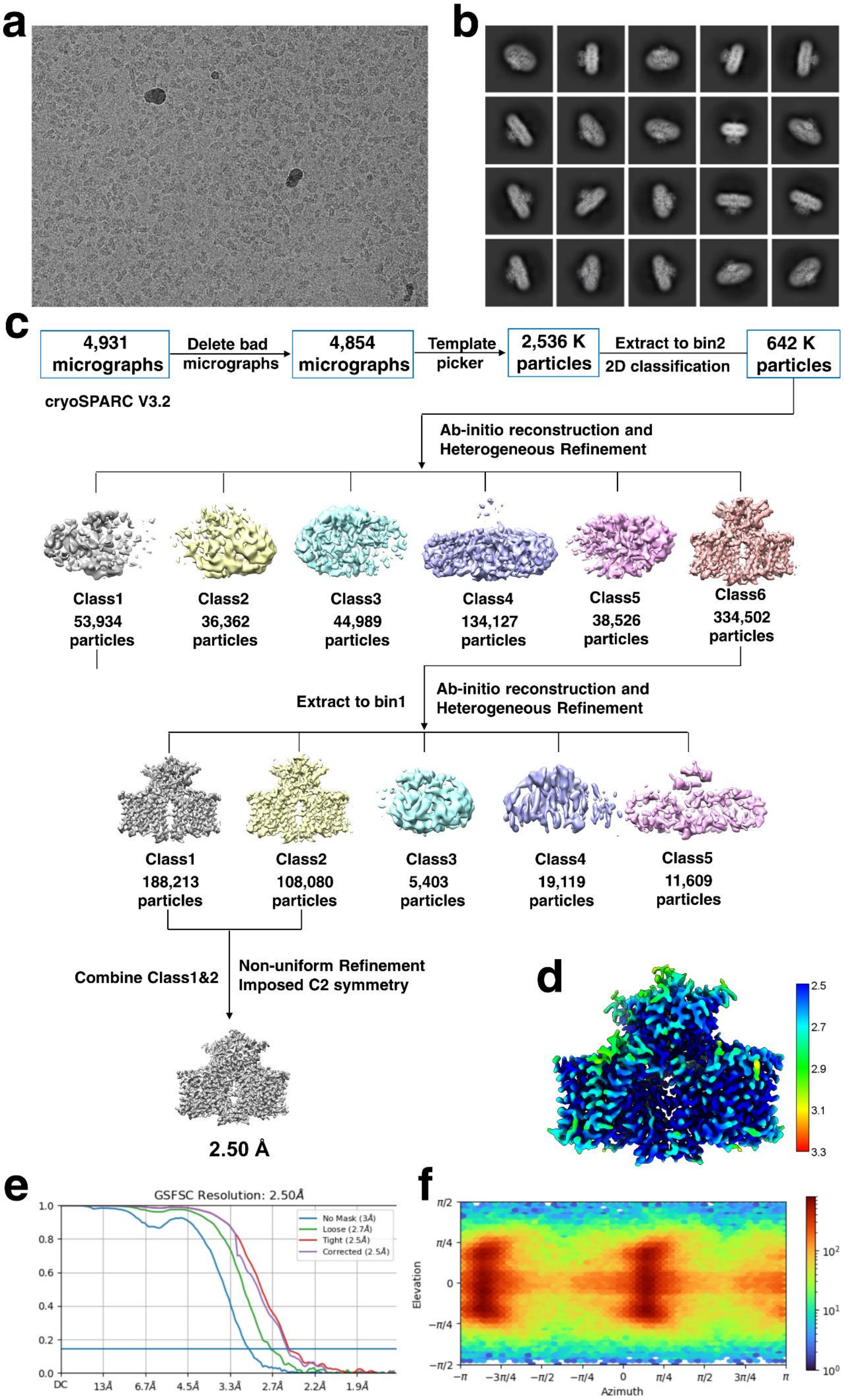
a
b
c
4,931 micrographs
Delete bad micrographs
4,854 micrographs
Template picker
2,536 K particles
Extract to bin2
2D classification
642 K particles
cryoSPARC V3.2
Ab-initio reconstruction and Heterogeneous Refinement
Class1 53,934 particles
Class2 36,362 particles
Class3 44,989 particles
Class4 134,127 particles
Class5 38,526 particles
Class6 334,502 particles
Extract to bin1
Ab-initio reconstruction and Heterogeneous Refinement
Class1 188,213 particles
Class2 108,080 particles
Class3 5,403 particles
Class4 19,119 particles
Class5 11,609 particles
Combine Class1&2
Non-uniform Refinement
Imposed C2 symmetry
2.50 Å
d
2.5
2.7
2.9
3.1
3.3
e
GSFSC Resolution: 2.50Å
No Mask (3Å)
Loose (2.7Å)
Tight (2.5Å)
Corrected (2.5Å)
1.0
0.8
0.6
0.4
0.2
0.0
DC
13Å
6.7Å
4.5Å
3.3Å
2.7Å
2.2Å
1.9Å
f
Elevation
Azimuth
π/2
π/4
0
−π/4
−π/2
−π
−3π/4
−π/2
−π/4
0
π/4
π/2
3π/4
π
10^2
10^1
10^0

Extended Data Fig. 6 Reconstruction and structure determination of human BTR1 with Na^+ and H_3BO_3 in natural condition.

a,b Representative cryo-EM micrograph (**a**) and 2D class averages (**b**) of human BTR1 with Na^+ and H_3BO_3 in natural condition.

c, The workflow of cryo-EM data processing by cryoSPARC[6].

d, Local resolution map of the final 3D density map.

e, Gold-standard Fourier Shell correlation (FSC) curve after 3D refinement. The resolution estimation was based on the criterion of FSC 0.143 cutoff.

f, Particle orientation distributions in the last iteration of the structural refinement.

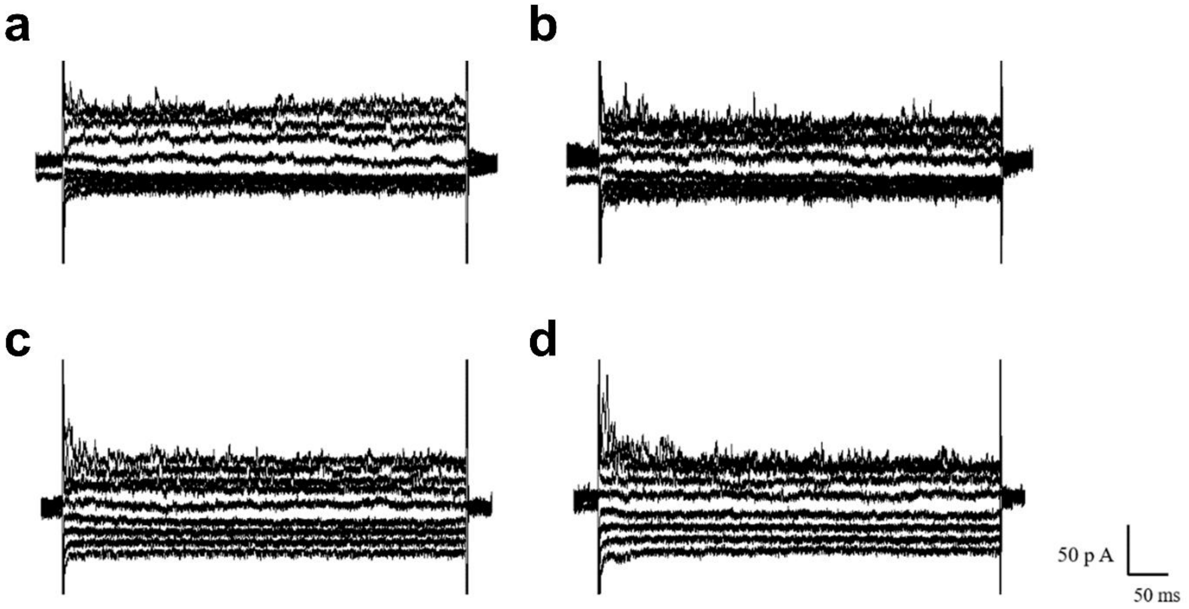

Extended Data Fig. 7 BTR1 is not boric acid transporter.

a-d, The patch-clamp recordings for macroscopic current in naïve HEK293T cells without boric acid (**a**), naïve HEK293T cells with boric acid (**b**), HEK293T cells overexpressed BTR1 without boric acid (**c**) and HEK293T cells overexpressed BTR1 with boric acid (**d**).

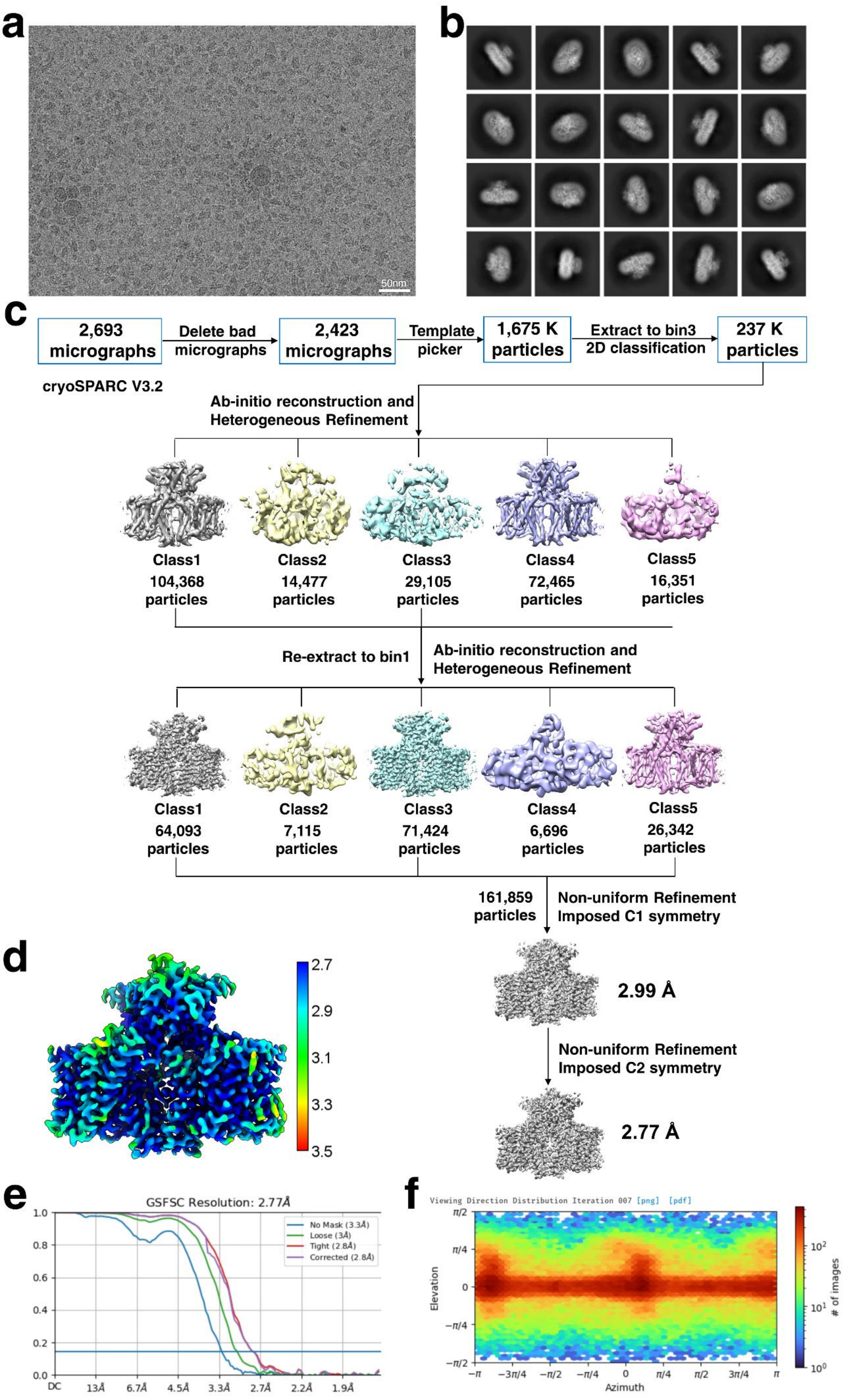
a
50nm
b
c
2,693 micrographs
Delete bad micrographs
2,423 micrographs
Template picker
1,675 K particles
Extract to bin3
2D classification
237 K particles
cryoSPARC V3.2
Ab-initio reconstruction and Heterogeneous Refinement
Class1 104,368 particles
Class2 14,477 particles
Class3 29,105 particles
Class4 72,465 particles
Class5 16,351 particles
Re-extract to bin1
Ab-initio reconstruction and Heterogeneous Refinement
Class1 64,093 particles
Class2 7,115 particles
Class3 71,424 particles
Class4 6,696 particles
Class5 26,342 particles
161,859 particles
Non-uniform Refinement
Imposed C1 symmetry
2.99 Å
Non-uniform Refinement
Imposed C2 symmetry
2.77 Å
d
2.7
2.9
3.1
3.3
3.5
e
GSFSC Resolution: 2.77Å
No Mask (3.3Å)
Loose (3Å)
Tight (2.8Å)
Corrected (2.8Å)
DC
13Å
6.7Å
4.5Å
3.3Å
2.7Å
2.2Å
1.9Å
f
Viewing Direction Distribution Iteration 007 [png] [pdf]
Elevation
Azimuth
# of images

Extended Data Fig. 8 Reconstruction and structure determination of human BTR1 without Na^+ in acidic condition.

a,b Representative cryo-EM micrograph (**a**) and 2D class averages (**b**) of human BTR1 without Na^+ in acidic condition.

c, The workflow of cryo-EM data processing by cryoSPARC[6].

d, Local resolution map of the final 3D density map.

e, Gold-standard Fourier Shell correlation (FSC) curve after 3D refinement. The resolution estimation was based on the criterion of FSC 0.143 cutoff.

f, Particle orientation distributions in the last iteration of the structural refinement.

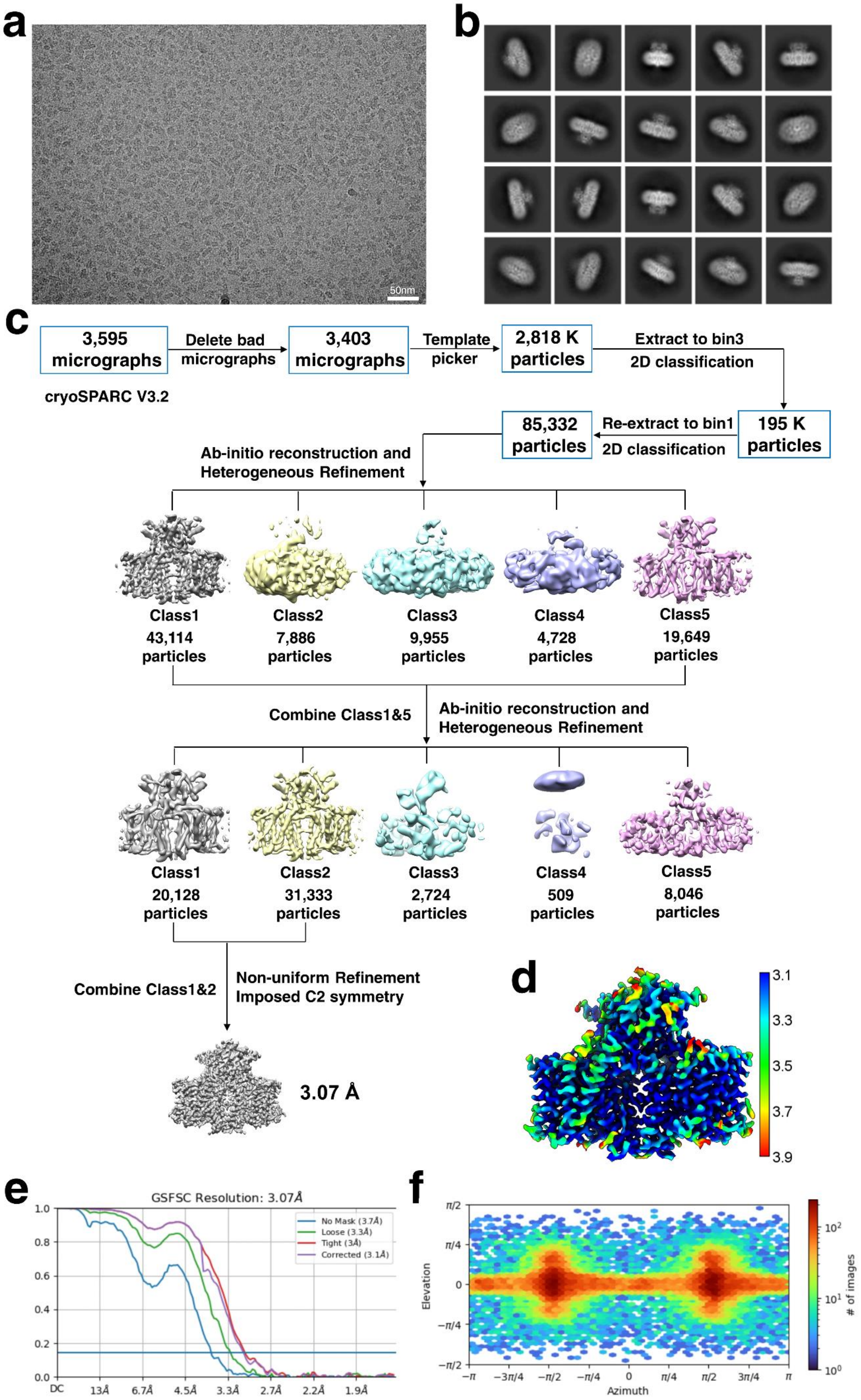
a
50nm
b
c
3,595 micrographs
Delete bad micrographs
3,403 micrographs
Template picker
2,818 K particles
Extract to bin3
2D classification
cryoSPARC V3.2
85,332 particles
Re-extract to bin1
2D classification
195 K particles
Ab-initio reconstruction and Heterogeneous Refinement
Class1 43,114 particles
Class2 7,886 particles
Class3 9,955 particles
Class4 4,728 particles
Class5 19,649 particles
Combine Class1&5
Ab-initio reconstruction and Heterogeneous Refinement
Class1 20,128 particles
Class2 31,333 particles
Class3 2,724 particles
Class4 509 particles
Class5 8,046 particles
Combine Class1&2
Non-uniform Refinement
Imposed C2 symmetry
3.07 Å
d
3.1
3.3
3.5
3.7
3.9
e
GSFSC Resolution: 3.07Å
No Mask (3.7Å)
Loose (3.3Å)
Tight (3Å)
Corrected (3.1Å)
1.0
0.8
0.6
0.4
0.2
0.0
DC
13Å
6.7Å
4.5Å
3.3Å
2.7Å
2.2Å
1.9Å
f
π/2
π/4
0
−π/4
−π/2
Elevation
−π
−3π/4
−π/2
−π/4
0
π/4
π/2
3π/4
π
Azimuth
10²
10¹
10⁰
# of images

Extended Data Fig. 9 Reconstruction and structure determination of human BTR1 with Na^+ in natural condition.

a,b Representative cryo-EM micrograph (**a**) and 2D class averages (**b**) of human BTR1 with Na^+ in natural condition.

c, The workflow of cryo-EM data processing by cryoSPARC[6].

d, Local resolution map of the final 3D density map.

e, Gold-standard Fourier Shell correlation (FSC) curve after 3D refinement. The resolution estimation was based on the criterion of FSC 0.143 cutoff.

f, Particle orientation distributions in the last iteration of the structural refinement.

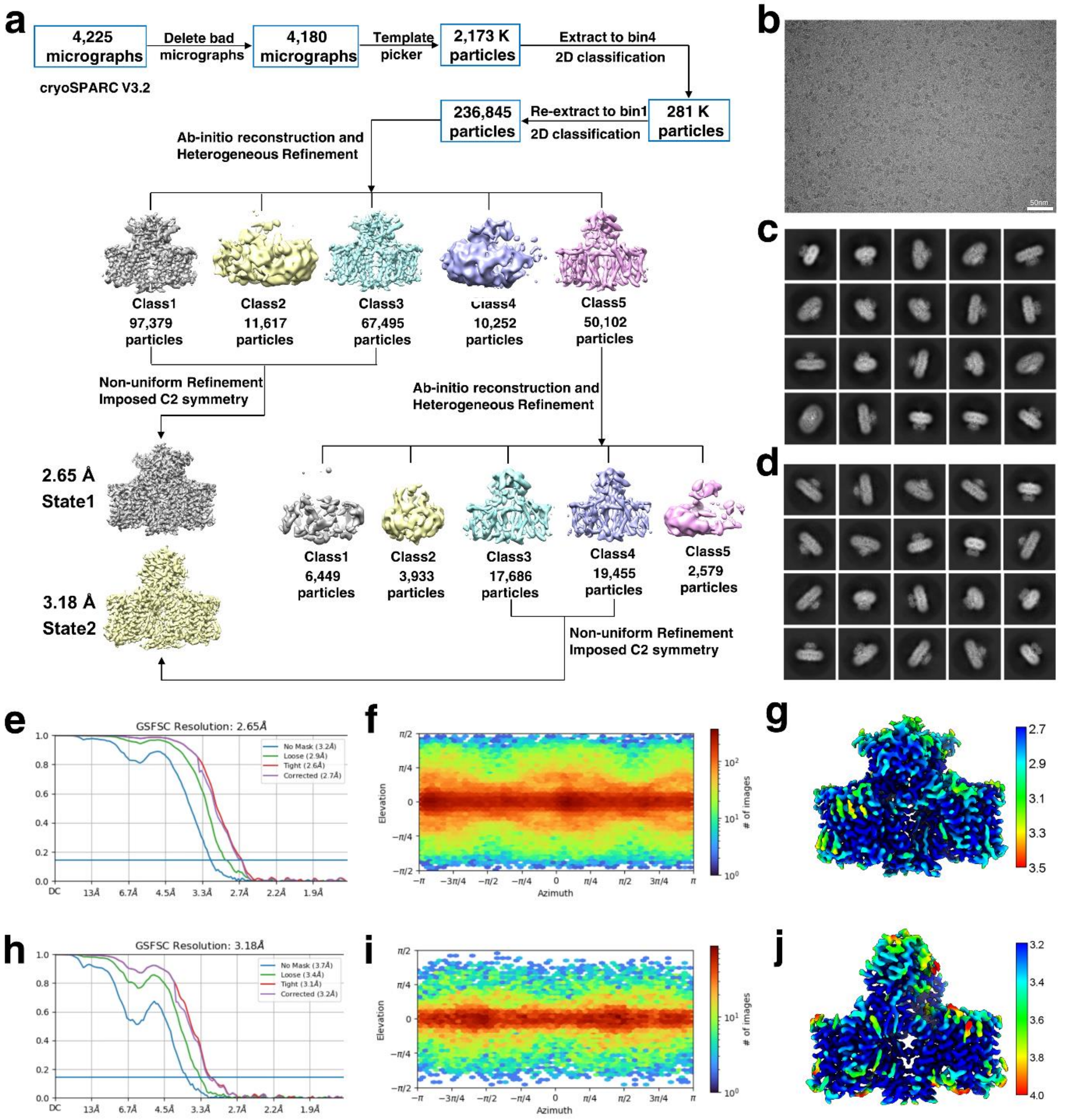

Extended Data Fig. 10 Reconstruction and structure determination of human BTR1 with Na^+ in acidic condition.

a, The workflow of cryo-EM data processing by cryoSPARC[6].

b Representative cryo-EM micrograph of human BTR1 with Na^+ in acidic condition.

c,d, Representative 2D average of the two BTR1 conformations in this dataset, state1 (**c**) and state2 (**d**).

e, Gold-standard Fourier Shell correlation (FSC) curve after 3D refinement of state1. The resolution estimation was based on the criterion of FSC 0.143 cutoff.

f, Particle orientation distributions in the last iteration of the structural refinement of state1.

g, Local resolution map of the final 3D density maps of state1.

h, Gold-standard Fourier Shell correlation (FSC) curve after 3D refinement of state2. The resolution estimation was based on the criterion of FSC 0.143 cutoff.

i, Particle orientation distributions in the last iteration of the structural refinement of state2.

j, Local resolution map of the final 3D density maps of state2.

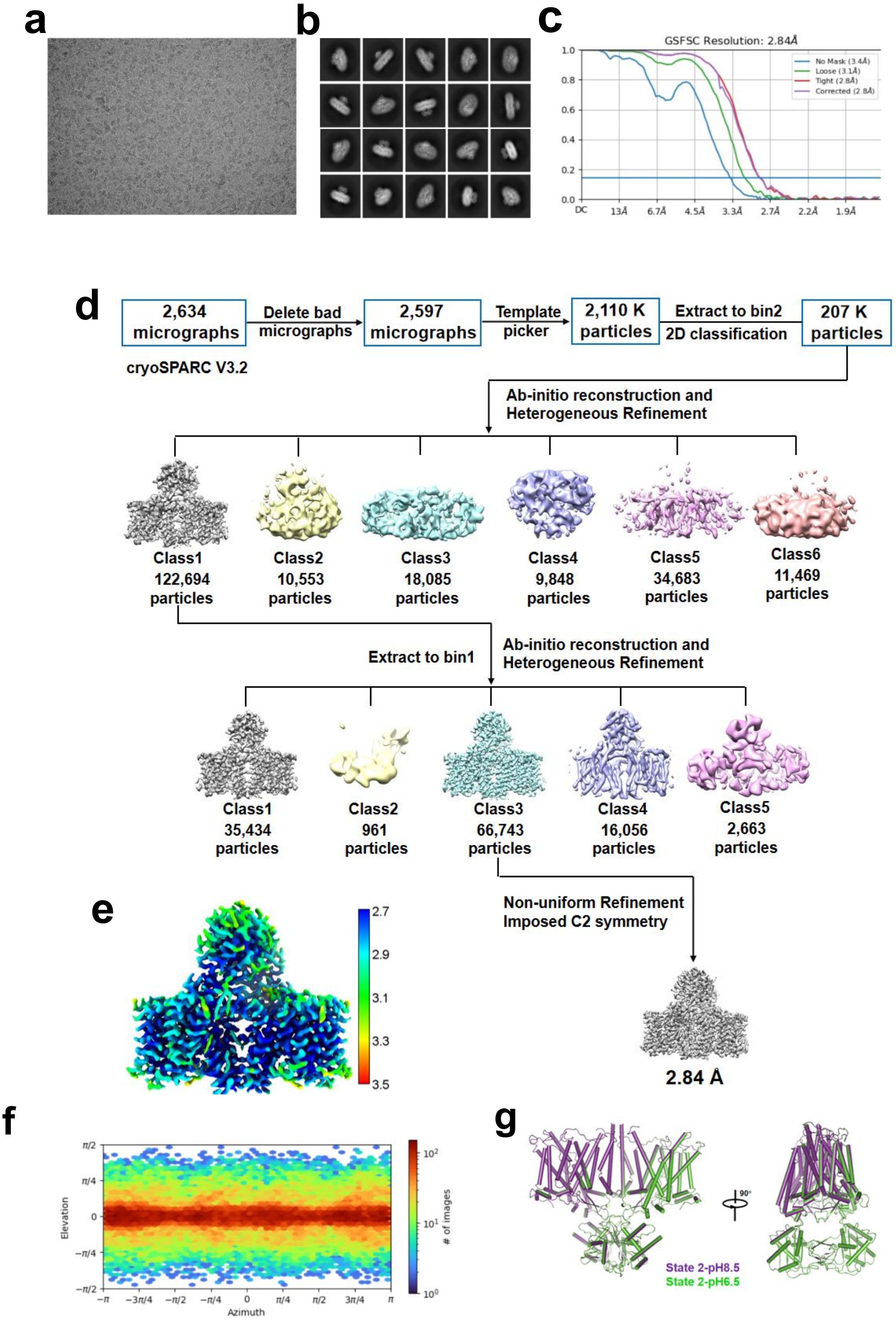
a
b
c
GSFSC Resolution: 2.84Å
No Mask (3.4Å)
Loose (3.1Å)
Tight (2.8Å)
Corrected (2.8Å)
DC
13Å
6.7Å
4.5Å
3.3Å
2.7Å
2.2Å
1.9Å
d
2,634 micrographs
cryoSPARC V3.2
Delete bad micrographs
2,597 micrographs
Template picker
2,110 K particles
Extract to bin2
2D classification
207 K particles
Ab-initio reconstruction and Heterogeneous Refinement
Class1 122,694 particles
Class2 10,553 particles
Class3 18,085 particles
Class4 9,848 particles
Class5 34,683 particles
Class6 11,469 particles
Extract to bin1
Ab-initio reconstruction and Heterogeneous Refinement
Class1 35,434 particles
Class2 961 particles
Class3 66,743 particles
Class4 16,056 particles
Class5 2,663 particles
Non-uniform Refinement
Imposed C2 symmetry
2.84 Å
e
2.7
2.9
3.1
3.3
3.5
f
Elevation
Azimuth
# of images
g
90°
State 2-pH8.5
State 2-pH6.5

Extended Data Fig. 11 Reconstruction and structure determination of human BTR1 with Na^+ in alkaline condition.

a,b Representative cryo-EM micrograph (**a**) and 2D class averages (**b**) of human BTR1 with Na^+ in alkaline condition.

c, The workflow of cryo-EM data processing by cryoSPARC[6].

d, Local resolution map of the final 3D density map.

e, Gold-standard Fourier Shell correlation (FSC) curve after 3D refinement. The resolution estimation was based on the criterion of FSC 0.143 cutoff.

f, Particle orientation distributions in the last iteration of the structural refinement.

g, Structural comparsions of the overall structures of actavition states at pH6.5 and pH8.5.

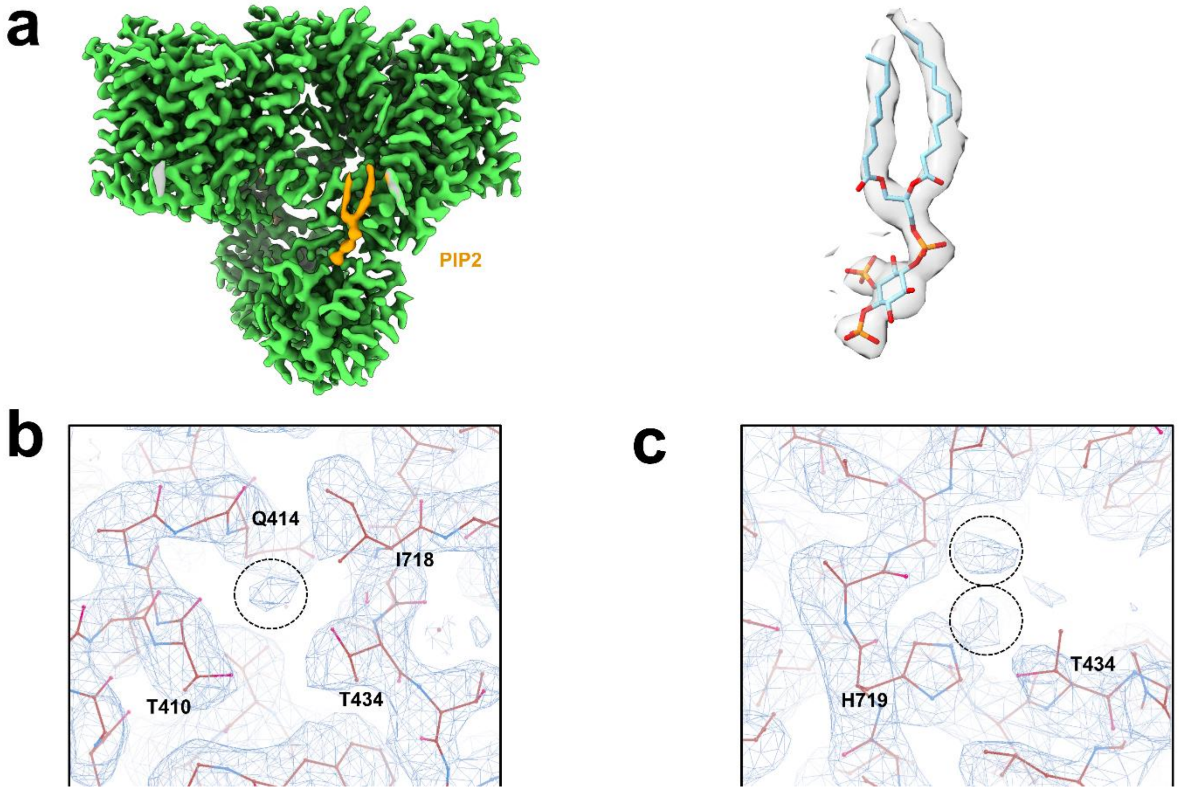

Extended Data Fig. 12 PIP2 and potential substrate density sites in BTR1

a, Position of PIP2 and corresponding density in the outward-open conformation;

b, Potential substrate-binding density around residues T410, T434, and Q414;

c, Potential substrate-binding density around residues H719 and T434.

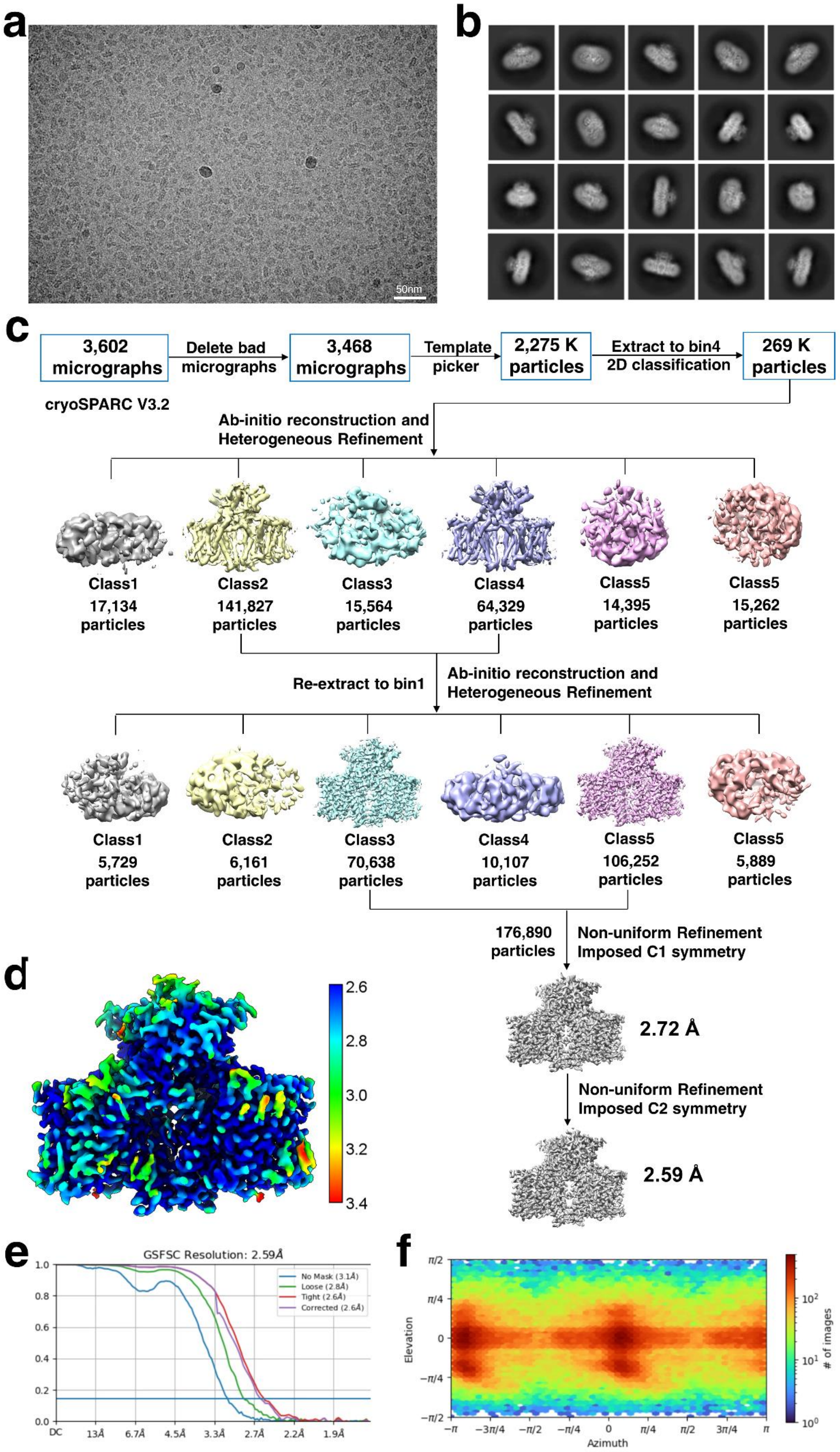
a
50nm
b
c
3,602 micrographs
Delete bad micrographs
3,468 micrographs
Template picker
2,275 K particles
Extract to bin4 2D classification
269 K particles
cryoSPARC V3.2
Ab-initio reconstruction and Heterogeneous Refinement
Class1 17,134 particles
Class2 141,827 particles
Class3 15,564 particles
Class4 64,329 particles
Class5 14,395 particles
Class5 15,262 particles
Re-extract to bin1
Ab-initio reconstruction and Heterogeneous Refinement
Class1 5,729 particles
Class2 6,161 particles
Class3 70,638 particles
Class4 10,107 particles
Class5 106,252 particles
Class5 5,889 particles
176,890 particles
Non-uniform Refinement Imposed C1 symmetry
2.72 Å
Non-uniform Refinement Imposed C2 symmetry
2.59 Å
d
2.6
2.8
3.0
3.2
3.4
e
GSFSC Resolution: 2.59Å
No Mask (3.1Å)
Loose (2.8Å)
Tight (2.6Å)
Corrected (2.6Å)
1.0
0.8
0.6
0.4
0.2
0.0
DC
13Å
6.7Å
4.5Å
3.3Å
2.7Å
2.2Å
1.9Å
f
π/2
π/4
0
−π/4
−π/2
Elevation
−π
−3π/4
−π/2
−π/4
0
π/4
π/2
3π/4
π
Azimuth
10^2
10^1
10^0
# of images

Extended Data Fig. 13 Reconstruction and structure determination of human BTR1 with NH_3 but without Na^+ in acidic condition.

a,b Representative cryo-EM micrograph (**a**) and 2D class averages (**b**) of human BTR1 with NH_3 but without Na^+ in acidic condition.

c, The workflow of cryo-EM data processing by cryoSPARC[6].

d, Local resolution map of the final 3D density map.

e, Gold-standard Fourier Shell correlation (FSC) curve after 3D refinement. The resolution estimation was based on the criterion of FSC 0.143 cutoff.

f, Particle orientation distributions in the last iteration of the structural refinement.

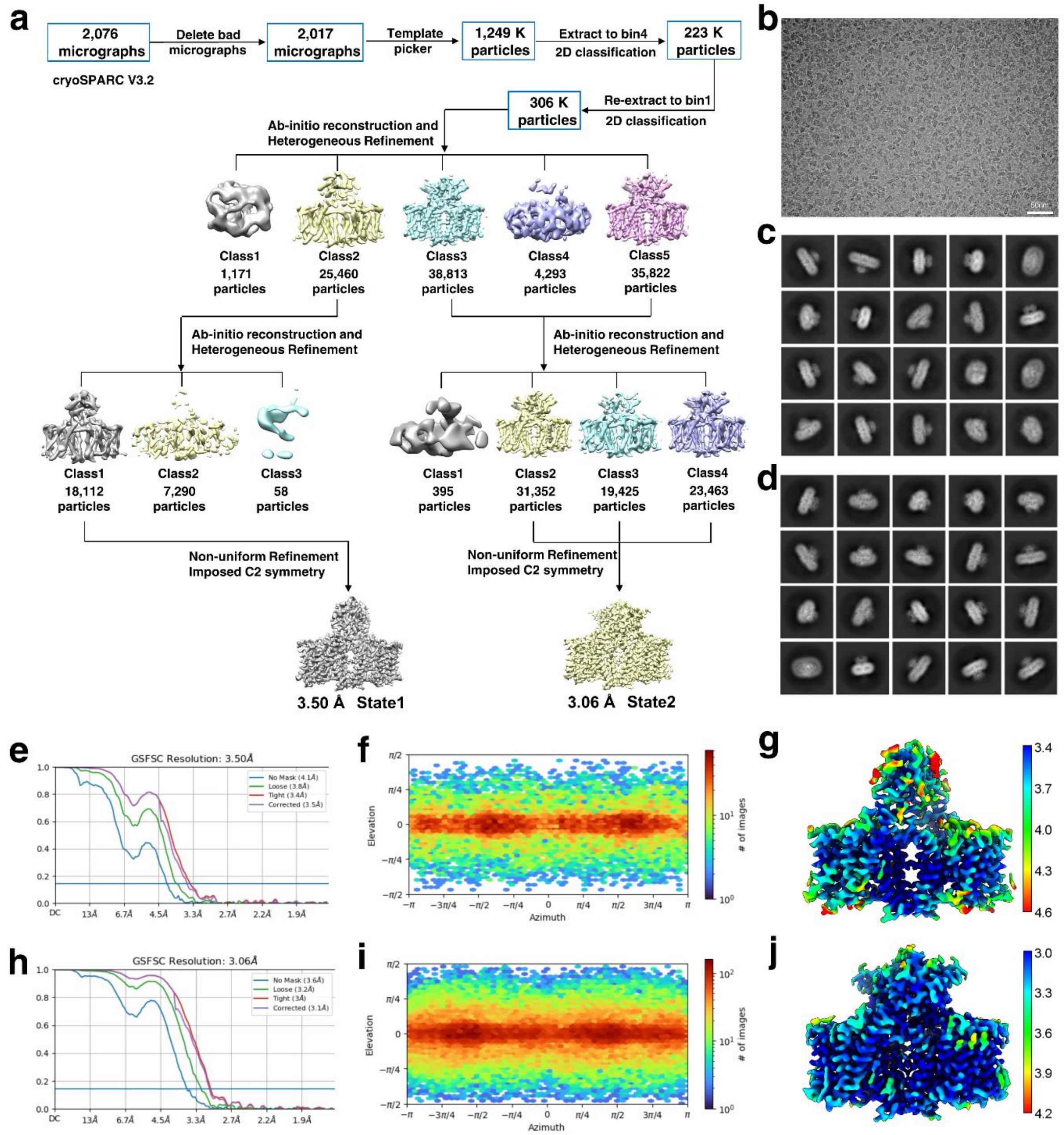

Extended Data Fig. 14 Reconstruction and structure determination of human BTR1 with NH_3 but without Na^+ in natural condition.

a, The workflow of cryo-EM data processing by cryoSPARC[6].

b Representative cryo-EM micrograph of human BTR1 with NH_3 but without Na^+ in natural condition.

c,d, Representative 2D average of the two BTR1 conformations in this dataset, state1 (**c**) and state2 (**d**).

e, Gold-standard Fourier Shell correlation (FSC) curve after 3D refinement of state1. The resolution estimation was based on the criterion of FSC 0.143 cutoff.

f, Particle orientation distributions in the last iteration of the structural refinement of state1.

g, Local resolution map of the final 3D density maps of state1.

h, Gold-standard Fourier Shell correlation (FSC) curve after 3D refinement of state2. The resolution estimation was based on the criterion of FSC 0.143 cutoff.

i, Particle orientation distributions in the last iteration of the structural refinement of state2.

j, Local resolution map of the final 3D density maps of state2.

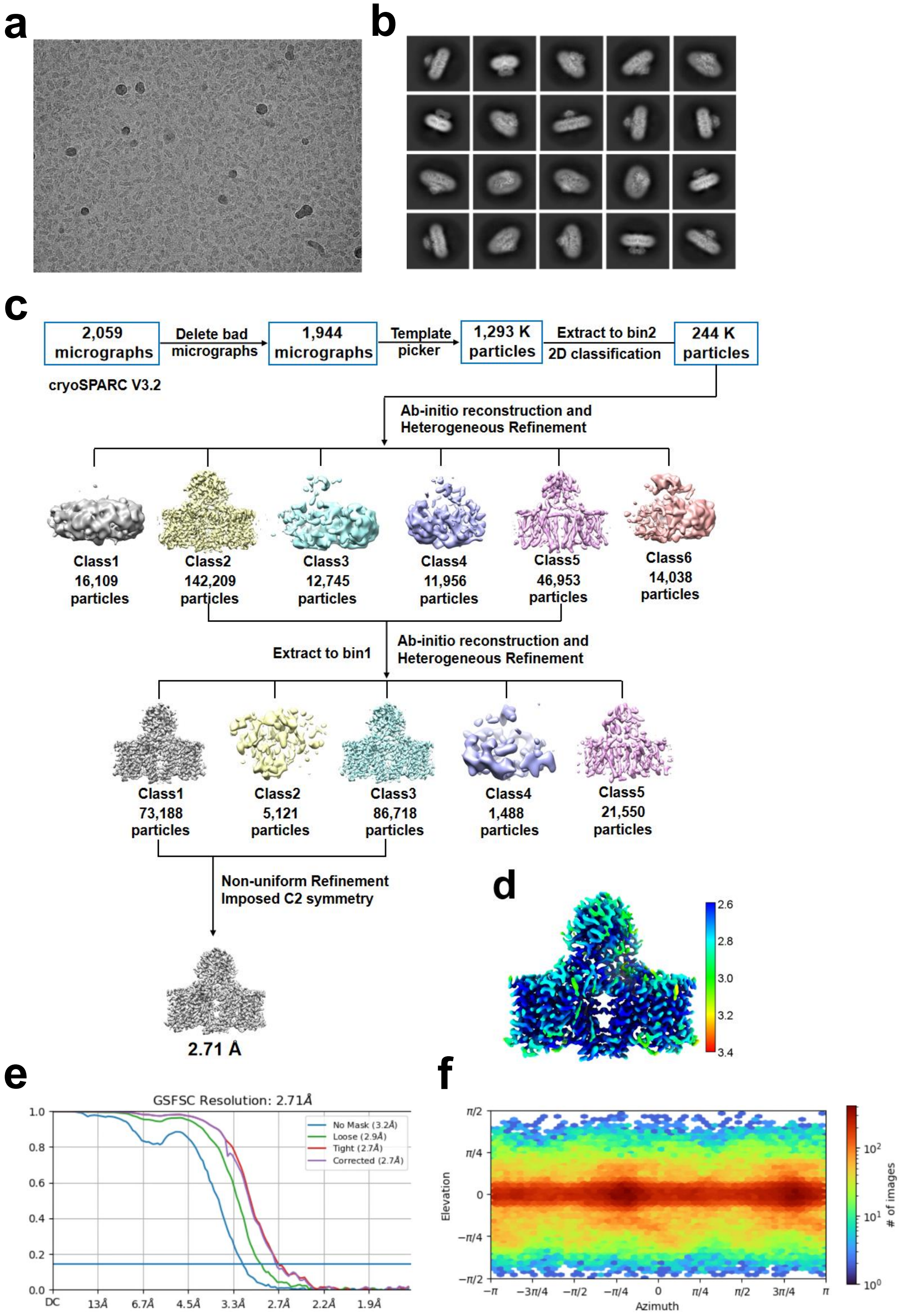
a
b
c
2,059 micrographs
Delete bad micrographs
1,944 micrographs
Template picker
1,293 K particles
Extract to bin2
2D classification
244 K particles
cryoSPARC V3.2
Ab-initio reconstruction and Heterogeneous Refinement
Class1 16,109 particles
Class2 142,209 particles
Class3 12,745 particles
Class4 11,956 particles
Class5 46,953 particles
Class6 14,038 particles
Extract to bin1
Ab-initio reconstruction and Heterogeneous Refinement
Class1 73,188 particles
Class2 5,121 particles
Class3 86,718 particles
Class4 1,488 particles
Class5 21,550 particles
Non-uniform Refinement
Imposed C2 symmetry
2.71 Å
d
2.6
2.8
3.0
3.2
3.4
e
GSFSC Resolution: 2.71Å
No Mask (3.2Å)
Loose (2.9Å)
Tight (2.7Å)
Corrected (2.7Å)
1.0
0.8
0.6
0.4
0.2
0.0
DC
13Å
6.7Å
4.5Å
3.3Å
2.7Å
2.2Å
1.9Å
f
Elevation
π/2
π/4
0
−π/4
−π/2
−π
−3π/4
−π/2
−π/4
0
π/4
π/2
3π/4
π
Azimuth
# of images
10²
10¹
10⁰

Extended Data Fig. 15 Reconstruction and structure determination of human BTR1 with NH_3 but without Na^+ in alkaline condition.

a,b Representative cryo-EM micrograph (**a**) and 2D class averages (**b**) of human BTR1 with NH_3 but without Na^+ in alkaline condition.

c, The workflow of cryo-EM data processing by cryoSPARC[6].

d, Local resolution map of the final 3D density map.

e, Gold-standard Fourier Shell correlation (FSC) curve after 3D refinement. The resolution estimation was based on the criterion of FSC 0.143 cutoff.

f, Particle orientation distributions in the last iteration of the structural refinement.

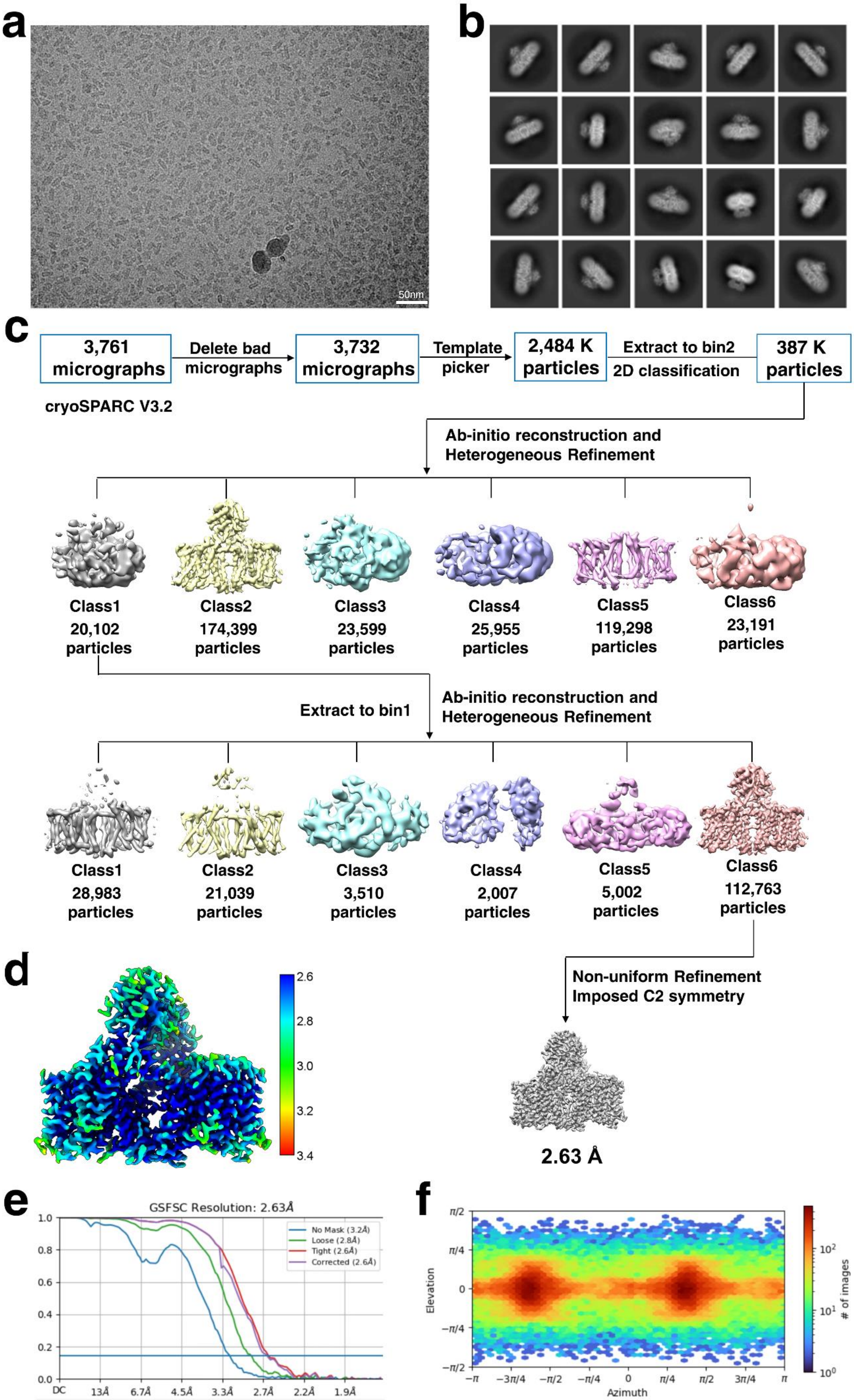
a
50nm
b
c
3,761 micrographs
Delete bad micrographs
3,732 micrographs
Template picker
2,484 K particles
Extract to bin2
2D classification
387 K particles
cryoSPARC V3.2
Ab-initio reconstruction and Heterogeneous Refinement
Class1 20,102 particles
Class2 174,399 particles
Class3 23,599 particles
Class4 25,955 particles
Class5 119,298 particles
Class6 23,191 particles
Extract to bin1
Ab-initio reconstruction and Heterogeneous Refinement
Class1 28,983 particles
Class2 21,039 particles
Class3 3,510 particles
Class4 2,007 particles
Class5 5,002 particles
Class6 112,763 particles
Non-uniform Refinement
Imposed C2 symmetry
2.63 Å
d
2.6
2.8
3.0
3.2
3.4
e
GSFSC Resolution: 2.63Å
No Mask (3.2Å)
Loose (2.8Å)
Tight (2.6Å)
Corrected (2.6Å)
1.0
0.8
0.6
0.4
0.2
0.0
DC
13Å
6.7Å
4.5Å
3.3Å
2.7Å
2.2Å
1.9Å
f
Elevation
Azimuth
# of images

Extended Data Fig. 16 Reconstruction and structure determination of human BTR1 with NH_3 and Na^+ in natural condition.

a,b Representative cryo-EM micrograph (**a**) and 2D class averages (**b**) of human BTR1 with NH_3 and Na^+ in natural condition.

c, The workflow of cryo-EM data processing by cryoSPARC[6].

d, Local resolution map of the final 3D density map.

e, Gold-standard Fourier Shell correlation (FSC) curve after 3D refinement. The resolution estimation was based on the criterion of FSC 0.143 cutoff.

f, Particle orientation distributions in the last iteration of the structural refinement.

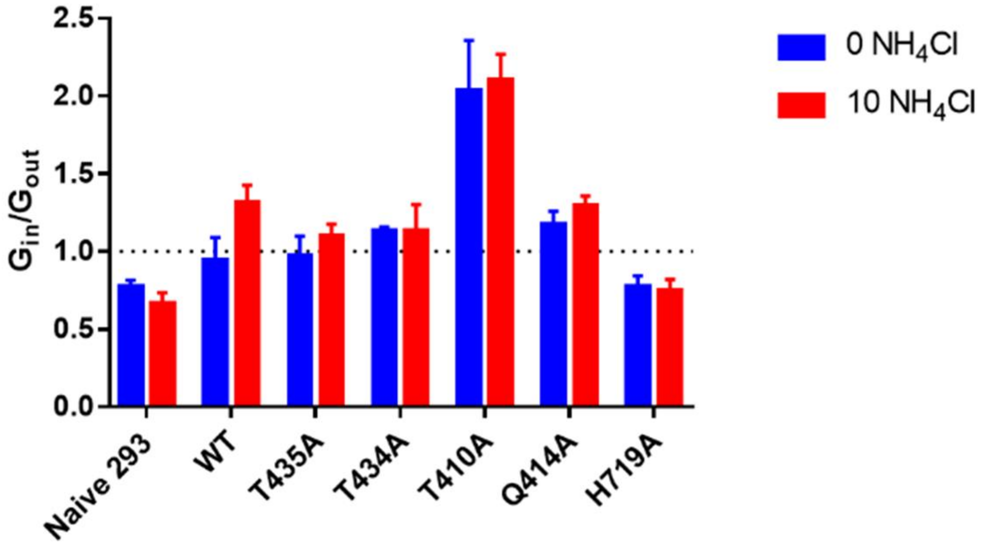

Extended Data Fig. 17 Several BTR1 mutations affect the ratio of the electrical conductance inward current to outward current (G_{in}/G_{out})

The $\mathbf{G_{in}/G_{out}}$ ratio in the naïve HEK293T cells or HEK293T cell overexpress BTR1 (WT or mutations) in the presence or absence of 10 mM NH_4Cl.

Reference

Adams, P.D., Grosse-Kunstleve, R.W., Hung, L.W., Ioerger, T.R., and Terwilliger, T.C. (2002). PHENIX: building new software for automated crystallographic structure determination. Acta Crystallographica Section D Biological Crystallography 58, 1948-1954.

Case, D.A., Ben-Shalom, I.Y., Brozell, S.R., Cerutti, D.S., and Kollman, P.A. (2018). Amber 2018.

Emsley, P., and Cowtan, K. (2004). Coot: model-building tools for molecular graphics. Acta Crystallogr D Biol Crystallogr 60, 2126-2132.

Emsley, P., Lohkamp, B., Scott, W.G., and Cowtan, K. (2010). Features and development of Coot. Acta Crystallographica Section D: Biological Crystallography 66.

Eric, Pettersen, Thomas, Goddard, Conrad, Huang, and Gregory (2008). UCSF Chimera--a visualization system for exploratory research and analysis. Journal of Computational Chemistry.

Goddard, T.D., Huang, C.C., Meng, E.C., Pettersen, E.F., Couch, G.S., Morris, J.H., and Ferrin, T.E. (2017). UCSF ChimeraX: Meeting modern challenges in visualization and analysis. Protein Science.

Jorgensen, W.L., Chandrasekhar, J., Madura, J.D., Impey, R., and Klein, M.L. (1983). Comparison of simple potential functions for simulating liquid water. Journal of Chemical Physics 79, 926-935.

Jumper, J., Evans, R., Pritzel, A., Green, T., and Hassabis, D. (2021). Highly accurate protein structure prediction with AlphaFold. Nature, 1-11.

Lei, J., and Frank, J. (2005). Automated acquisition of cryo-electron micrographs for single particle reconstruction on an FEI Tecnai electron microscope. Journal of Structural Biology 150, 69-80.

Punjani, A., Rubinstein, J.L., Fleet, D.J., and Brubaker, M.A. (2017). cryoSPARC: algorithms for rapid unsupervised cryo-EM structure determination. Nature Methods 14, 290-296.

Punjani, A., Zhang, H., and Fleet, D.J. (2020). Non-uniform refinement: adaptive regularization improves single-particle cryo-EM reconstruction. Nature Methods 17, 1214-1221.

Simmerling, Carlos, Hauser, Kevin, E., Maier, James, A., Kasavajhala, and Koushik (2015).

ff14SB: Improving the Accuracy of Protein Side Chain and Backbone Parameters from ff99SB. Journal of Chemical Theory & Computation Jctc.

Zheng, S.Q., Palovcak, E., Armache, J.P., Verba, K.A., Cheng, Y., and Agard, D.A. MotionCor2: anisotropic correction of beam-induced motion for improved cryo-electron microscopy. Nature Methods.
